# Structural basis of a small monomeric Clivia fluorogenic RNA with a large Stokes shift

**DOI:** 10.1038/s41589-024-01633-1

**Published:** 2024-05-30

**Authors:** Kaiyi Huang, Qianqian Song, Mengyue Fang, Deqiang Yao, Xin Shen, Xiaochen Xu, Xianjun Chen, Linyong Zhu, Yi Yang, Aiming Ren

**Affiliations:** 1 Department of Cardiology, The Second Affiliated Hospital of School of Medicine, Zhejiang University, Hangzhou, China; 2 Life Sciences Institute, Zhejiang University, Hangzhou, China; 3 Optogenetics and Synthetic Biology Interdisciplinary Research Center, State Key Laboratory of Bioreactor Engineering, Shanghai Frontiers Science Center of Optogenetic Techniques for Cell Metabolism, East China University of Science and Technology, Shanghai, China; 4 School of Pharmacy, East China University of Science and Technology, Shanghai, China; 5 Institute of Aging and Tissue Regeneration, Renji Hospital, School of Medicine, Shanghai Jiao Tong University, Shanghai, China; 6 School of Biomedical Engineering, Shanghai Jiao Tong University, Shanghai, China

**Keywords:** RNA, RNA, X-ray crystallography

## Abstract

RNA-based fluorogenic modules have revolutionized the spatiotemporal localization of RNA molecules. Recently, a fluorophore named 5-((*Z*)-4-((2-hydroxyethyl)(methyl)amino)benzylidene)-3-methyl-2-((*E*)-styryl)-3,5-dihydro-4*H*-imidazol-4-one (NBSI), emitting in red spectrum, and its cognate aptamer named Clivia were identified, exhibiting a large Stokes shift. To explore the underlying molecular basis of this unique RNA–fluorophore complex, we determined the tertiary structure of Clivia–NBSI. The overall structure uses a monomeric, non-G-quadruplex compact coaxial architecture, with NBSI sandwiched at the core junction. Structure-based fluorophore recognition pattern analysis, combined with fluorescence assays, enables the orthogonal use of Clivia–NBSI and other fluorogenic aptamers, paving the way for both dual-emission fluorescence and bioluminescence imaging of RNA molecules within living cells. Furthermore, on the basis of the structure-based substitution assay, we developed a multivalent Clivia fluorogenic aptamer containing multiple minimal NBSI-binding modules. This innovative design notably enhances the recognition sensitivity of fluorophores both in vitro and in vivo, shedding light on future efficient applications in various biomedical and research contexts.

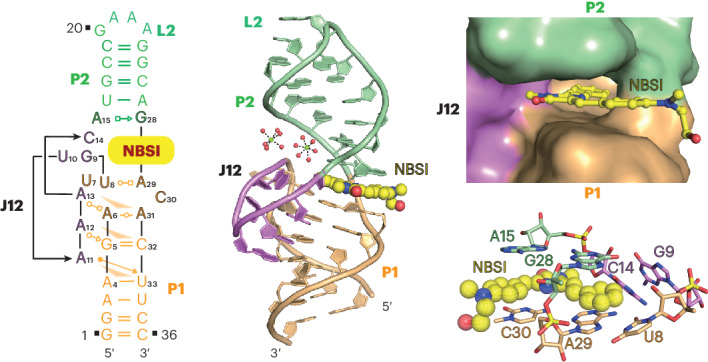

## Abstract (web summary)

Huang et al. report the tertiary structure of a small monomeric fluorogenic RNA aptamer named Clivia, characterized by a large Stokes shift, revealing the fluorescence activation mechanism and enabling a multivalent design to enhance the fluorescence output at specific dye concentrations.

## Main

The emergence of intrinsically fluorescent proteins (FPs), notably green FP (GFP), has brought about a revolutionary advancement in the visualization of proteins, both in vivo and in vitro, greatly propelling proteome studies^1^. While the critical roles of various RNA molecules within diverse biological systems are well acknowledged, the discovery of intrinsically fluorescent RNA molecules has remained elusive. Consequently, the accurate spatiotemporal localization of RNA molecules is still a challenge, hindered by the limitations of the prevailing RNA imaging technologies (fluorescence in situ hybridization (FISH)^2,3^, the MS2 coat protein (MCP)–FP system^4^, etc.^5^). FISH is able to capture static images only after the cell fixation step. The MCP–FP system allows spatiotemporal imaging of RNA molecules in living cells but is interfered with by a high background of unbound FP proteins.

To address these hurdles, RNA-based fluorogenic aptamers have been developed through systematic evolution of ligands by exponential enrichment (SELEX) technology to light up weakly or nonfluorescent small-molecule fluorophores. Early examples of aptamers and their cognate chromophores have been developed, including malachite green (MG) aptamer binding to tetramethylrosamine (TMR)^6,7^, Spinach and its variants activating the fluorescence of small-molecule analogs of the intrinsic chromophores of FPs^8–11^ and Mango RNA and reselected variants binding to dyes named thiazole orange derivatives^12–14^. However, issues such as the high fluorescence background of unbound fluorophores, the tendency of aptamer scaffolds containing a G-quadruplex to misfold, weak cell brightness and poor photostability^6,7,11,15^ have hindered their wide application. The recent development of non-G-quadruplex fluorogenic aptamers, dimethylindole red (DIR) and Pepper, was aimed at circumventing these challenges. However, certain limitations, such as a dimerization tendency and modest excitation–emission shifts, persist^16–19^. Fluorogenic modules with large Stokes shifts have notable advantages for convenient use^20^. One recently reported fluorogenic RNA aptamer named Chili emits with a large Stokes shift when bound to 3,5-dimethoxy-4-hydroxybenzylidene imidazolone^+^ (DMHBI^+^) analogs^21^. However, the low quantum yields (<0.1) of the Chili aptamer and the high fluorescence background of unbound dyes hinder its in vivo application.

The development and exploration of a broader range of fluorogenic RNA aptamers and cognate small-molecule fluorophores, characterized by diverse structures and improved photonic properties (in particular, a large Stokes shift), offer the potential to enable the simultaneous tracking of various RNA molecules, facilitating the use of both dual-emission fluorescence and bioluminescence imaging of RNA molecules within living cells. Ultimately, these efforts hold the promise of enriching and accelerating comprehensive transcriptome studies.

**Fig. 1:**
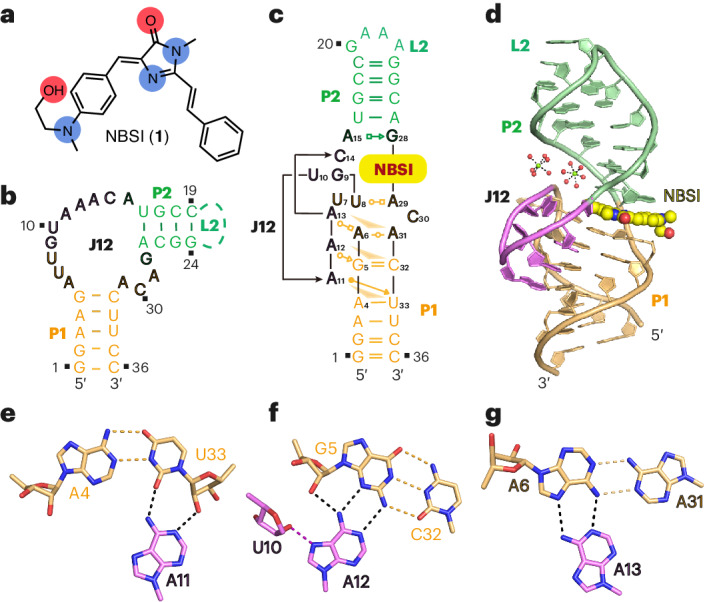
Structural topology and pairing alignments of the Clivia aptamer. **a**, Chemical structure of NBSI. **b**, Conserved sequence from SELEX screening and predicted secondary structure of the Clivia aptamer. The sequence is color-coded as in **d**. **c**, Schematic of the folding topology of the Clivia aptamer based on the tertiary structure. Additional base pairs are formed at the termini of stems P1 and P2. **d**, Cartoon representation of the tertiary structure of Clivia with ligand NBSI shown in ball representation. NBSI is located at the center of the overall structure and intercalates between two helical segments of the Clivia aptamer. Two fully hydrated Mg^2+^ (shown in ball representation) were identified between helix P2 and junction J12. **e**, A11 interacts with the sugar edge of U33 from the minor groove edge of one canonical base pair A4–U33. **f**, A12 interacts with G5 along its Watson–Crick edge from the minor groove edge of one canonical base pair G5–C32. The 2′-OH group of U10 forms a hydrogen bond with the N7 atom of A12. **g**, A6 forms a reverse Watson–Crick base pair with A31. A13 interacts with the Hoogsteen edge of A6 along its Watson–Crick edge.

NBSI (**1**) (chemical name 5-((*Z*)-4-((2-hydroxyethyl)(methyl)amino)benzylidene)-3-methyl-2-((*E*)-styryl)-3,5-dihydro-4*H*-imidazol-4-one; chemical structure in Fig. 1a) is a newly developed fluorophore molecule. The selected fluorogenic RNA to bind NBSI, named Clivia, is smaller than most current fluorogenic aptamers (Fig. 1b). Sequence analysis indicated that it has no G-quadruplex structure, which may overcome the misfolding tendency of some G-quadruplex fluorogenic aptamers^22^. Furthermore, NBSI exhibited low background fluorescence and high brightness upon binding to the Clivia aptamer. In vivo detection showed highly stable fluorescence of the Clivia–NBSI complex in living cells. Notably, the excitation and emission maxima of the NBSI–Clivia complex are 524 nm and 580 nm, respectively, which can be further tuned by modifying the fluorophore structure to achieve maximum Stokes shifts up to 108 nm (ref. ^22^).

To elucidate the underlying mechanism of how this small non-G-quadruplex RNA fluorogenic aptamer recognizes the cognate fluorophore and strongly enhances its fluorescence, we determined the tertiary structure of the Clivia–NBSI complex using X-ray crystallography. Structure-guided substitution assays identified the precise alignment of the residues accommodating the bound fluorophore molecule (NBSI) and its analogs. This revelation facilitated the tailoring of the original Clivia motif to the minimal Clivia fluorogenic module and assisted our design of the multivalent Clivia fluorogenic aptamer to enhance the fluorescence. Furthermore, we extended our exploration to the tertiary structure of the Clivia_III–NBSI complex, which contained three tandem arrays of Clivia fluorogenic modules. This thorough analysis validated and reinforced our design strategy, providing a robust structural foundation for the enhanced and more efficient use of the Clivia aptamer, both in vivo and in vitro.

## Results

### Tertiary structure of the Clivia–NBSI complex

As shown in Fig. 1b, the predicted secondary structure of Clivia contains two stems, P1 (orange) and P2 (green), that are connected by one big internal loop, J12 (purple). To facilitate the crystallization of the Clivia complex bound to NBSI, the variable apical loop L2 of stem P2 was replaced with the stable tetraloop or protein-binding loop (cocrystallization with the RNA-binding protein) (Fig. 1b), in which the GAAA tetraloop replacement generated crystals with high diffraction quality. The structure of the Clivia–NBSI complex was refined at a resolution of 1.6 Å with *R*_work_/*R*_free_ values of 0.19/0.22 (Supplementary Table 1). Each asymmetric unit contains two molecules of the Clivia–NBSI complex and minor molecular interactions were found between them (Supplementary Fig. 1a,b). Then, we used size-exclusion chromatography to detect the solution state of the Clivia–NBSI complex and found that it existed homogeneously as a monomer in solution (Supplementary Fig. 1c,d). Superposition of two molecules of the Clivia–NBSI complex in one asymmetric unit generated a root mean squared deviation (r.m.s.d.) value of 1.09 Å (ref. ^23^). To simplify the description, we focus on the structure of one molecule of the complex below. The contacts described below had a distance cutoff of 3.5 Å.

The tertiary fold of the Clivia–NBSI complex is shown schematically in Fig. 1c and its cartoon representation is shown in Fig. 1d. The overall structure of the complex adopts a coaxial long helix fold and is stabilized by continuous stacking interactions from the bottom stem P1 to the apical stem P2 with the ligand NBSI intercalated in the zipped junction region at the interface of stems P1 and P2 (Fig. 1c,d). A15 and G28 from the internal loop form a noncanonical base pair and elongate stem P2 adjacent to the terminal base pair U16–A27 of stem P2. A6 and A31 from the internal loop form another noncanonical base pair and stack on the northern top of stem P1. U8 pairs with A29 in the internal loop below the bound dye, NBSI. The consecutive nucleotides from G9 to C14 (shown in purple) bend downward to interact with the minor groove of stem P1 and form a stable multilayered platform for NBSI binding. The NBSI-binding pocket is located in the central junction of the overall structure between stems P1 and P2 (Fig. 1c,d).

### Stabilization interaction of the internal loop

**Extended Data Fig. 1:**
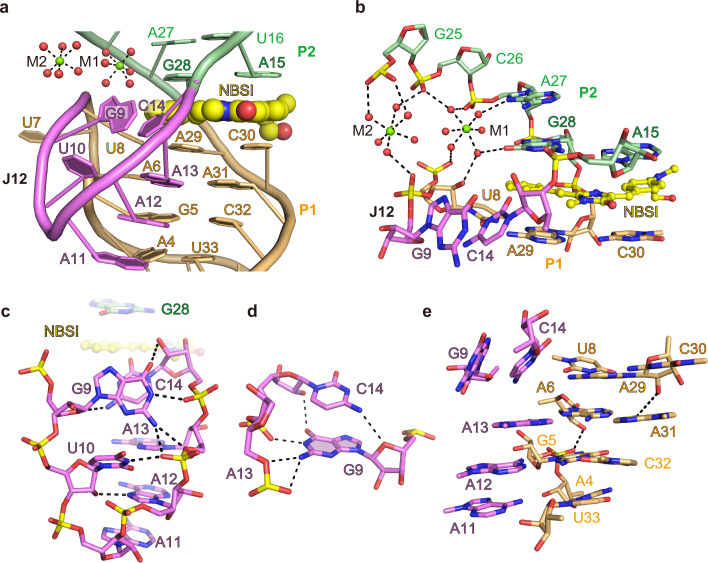
Junction interaction of Clivia aptamer. (**a**) The cartoon representation of the binding-site region of Clivia aptamer with ligand NBSI. Expanded view of panel highlighting M1 and M2 sites of Mol A, and the residues alignment of the junction. (**b**) The expended view of the stick representation of the binding-site region of Clivia aptamer with ligand NBSI. In addition to two fully hydrated Mg^2+^, one coordinated water molecule was also involved in the junction interaction. (**c**) The junction residues from G9 to C14 fold in a V-shaped conformation, in which compact interaction is formed between the base and the sugar-phosphate backbone. (**d**) G9 stacks partially with C14. While the base of G9 forms hydrogen-bonding interaction with the sugar-phosphate backbone of A13 and C14, 4-NH_2_ of C14 forms one hydrogen bond with O4′ of G9. (**e**) Consecutive tiers form by the junction residues and stack with each other above stem P1. Hydrogen-bonding interactions are also identified between different tiers. The sugar of C30 form hydrogen bond with the base of A31 and the sugar of A6 forms one hydrogen bond with the phosphate between G5 and A4.

Two fully hydrated Mg^2+^ ions (M1 and M2) were identified near the NBSI-binding pocket (Fig. 1d and Extended Data Fig. 1a), confirmed by the anomalous signal collected using Mn^2+^-soaked crystals (Supplementary Fig. 2). They form extensive hydrogen-bonding interactions with the sugar–phosphate backbone of stem P2 and the junction region, which may contribute to the stabilization of the NBSI-binding pocket (Extended Data Fig. 1b).

In addition to forming the noncanonical base pairs between stems P1 and P2, the consecutive nucleotides G9-U10-A11-A12-A13-C14 in internal loop J12 form a compact cap-like structure along the minor groove of stem P1 (Fig. 1c,d), in which U10-A11-A12-A13 forms continuous stacking interactions parallel to stem P1 and the two terminal residues G9 and C14 stack partially on each other in a direction tilted toward the long helix (Extended Data Fig. 1b,c). The Watson–Crick edge of G9 forms several hydrogen bonds with the 2′-OH group and the nonbridging phosphate oxygen of C14 and the bridging and nonbridging phosphate oxygens of A13 (Extended Data Fig. 1c,d). Furthermore, the 4-NH_2_ group of C14 forms another direct hydrogen bond with the 4′-O group of G9 (Extended Data Fig. 1c,d). It is notable that both G9 and C14 adopt a C2′-*endo* ribose sugar pucker conformation. The 2′-OH group of U10 forms a direct hydrogen bond with the N7 atom of A12 and the N3 atom of U10 forms another hydrogen bond with the nonbridging phosphate oxygen between A12 and A13 (Extended Data Fig. 1c).

A11, A12 and A13 stack on each other in the minor groove side of stem P1, consequently forming three consecutive tiers (Fig. 1c–g and Extended Data Fig. 1e). In the first tier, A11 interacts with the sugar edge of U33 along its Watson–Crick edge and forms an A4–U33·A11 base triple (Fig. 1e). In the second tier, A12 forms two hydrogen bonds with the base of G5 and one hydrogen bond with the 2′-OH group of G5. Additionally, the 2′-OH group of U10 was found to interact with the N7 atom of A12 in the same tier (Fig. 1f). The third tier is formed by three residues (A6, A13 and A31) from the internal loop, in which A6 forms a reverse base pair with A31 along their Watson–Crick edges and A13 interacts with the Hoogsteen edge of A6 from the minor groove side of stem P1 (Fig. 1g). It is notable that the ribose of A6 adopts a 2′-*endo* sugar pucker conformation in the structure (Fig. 1g). In addition, hydrogen-bonding interactions are found between different tiers (Extended Data Fig. 1e). The 2′-OH group of C30 in the first tier forms a hydrogen bond with the 6-NH_2_ group of A31 in the second tier. Additionally, the 2′-OH group of A6 in the second tier forms a hydrogen bond with the phosphate between A4 and G5 (Extended Data Fig. 1e).

### Composition of NBSI-binding pocket and recognition of NBSI

**Fig. 2:**
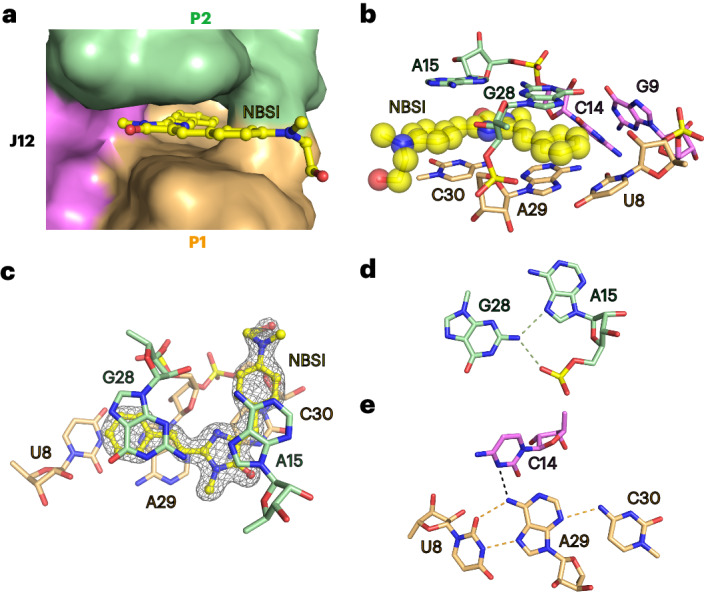
Structural alignment of NBSI-binding pocket of the Clivia aptamer. **a**, Surface representation of the Clivia aptamer bound to NBSI shown in stick representation. **b**, The planar moiety of ligand NBSI (shown in ball representation) is surrounded by three groups of consecutive junction residues U8-G9, C14-A15 and G28-A29-C30. A15 and G28 form one base pair and stack above NBSI. U8, A29 and C30 form one base triple and stack below NBSI. G9 and C14 stack on each other and bracket the side of NBSI. **c**, NBSI intercalates into the binding pocket and is sandwiched by two adjacent bases, in which the composite omit map of NBSI contoured at level 1.0*σ* is shown. **d**, The 2-NH_2_ group of G28 forms two hydrogen bonds with the N7 atom and nonbridging phosphate oxygen of A15. **e**, The Watson–crick edge of U8 forms a pairing interaction with the Hoogsteen edge of A29. The N3 atom of A29 forms an additional hydrogen bond with the 4-NH_2_ group of C30 and the 6-NH_2_ group of A29 forms an additional hydrogen bond with the N3 atom of C14.

The NBSI-binding pocket is located at the interface of stems P1 and P2 in the tertiary fold of the Clivia aptamer (shown in surface representation with NBSI shown in ball-and-stick representation; Fig. 2a). The major aromatic moieties of NBSI intercalate into the binding pocket, while the terminal (2-hydroxyethyl)(methyl)amino group of NBSI points outward from the binding pocket (Fig. 2a). Three groups of successive residues (C14-A15, U8-G9 and G28-A29-C30) from internal loop J12 constitute a three-sided box and encompass the bound NBSI in the binding pocket (Fig. 2b,c).

**Extended Data Fig. 2:**
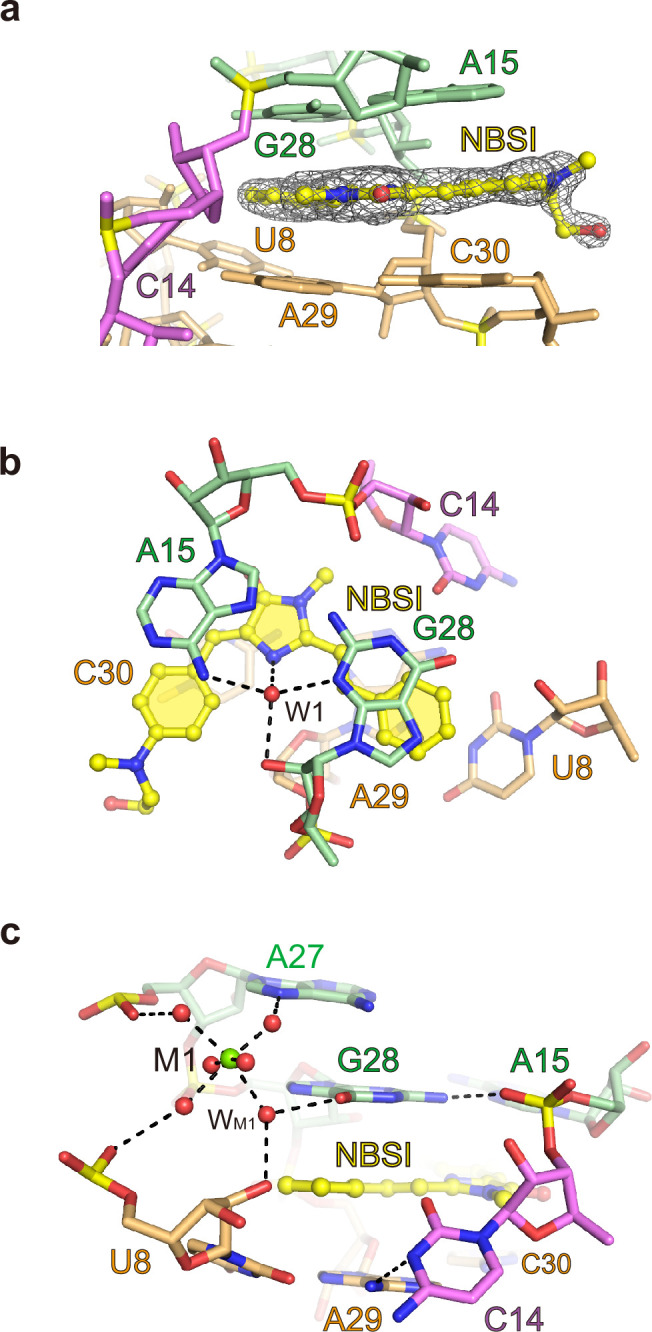
The residual interaction lining the binding pocket. (**a**) NBSI is stacked between two adjacent base tiers, in which the omit composite map (contoured at level 1.0 σ) of NBSI is shown as well. (**b**) One water molecule W1 mediates the interaction of NBSI and two stacked residues A15 and G28. (**c**) The fully hydrated Mg^2+^, M1 forms interaction with the base of G28, the phosphate and the base of A27, the sugar and the phosphate of U8 through coordinated water molecules. N3 of C14 forms hydrogen-bonding interaction with 6-NH_2_ of A29.

As shown in Fig. 2b,c and Extended Data Fig. 2a, NBSI is sandwiched between the upper two contacting bases A15·G28 and the lower base triple U8·A29·C30, anchored by the sugar–phosphate RNA backbone between G28 and A29 and capped by two adjacently stacked residues C14 and G9. The composite omit map of NBSI contoured at level 1.0*σ* is shown in Fig. 2c and Extended Data Fig. 2a. The 2-NH_2_ group of G28 forms a hydrogen bond with the N7 atom of A15 and a hydrogen bond with the nonbridging phosphate oxygen between A15 and C14 above the bound NBSI (Fig. 2d). The Hoogsteen edge of A29 forms a pairing interaction with the Watson–Crick edge of U8 and the N3 atom in the sugar edge of A29 forms another hydrogen bond with the 4-NH_2_ group of C30 below the bound NBSI. In addition, it was found that the 6-NH_2_ group of A29 forms a hydrogen bond with the N3 atom of the capped residue C14 (Fig. 2e). Here, U8 and A29 also adopt a 2′-*endo* sugar pucker conformation.

Even though no direct hydrogen-bonding interaction was identified between NBSI and the Clivia aptamer in the binding pocket, water molecule W1 was found to coordinate with the N1 atom of the imidazole moiety of NBSI and form extra hydrogen bonds with the neighboring residues, potentially facilitating the specific recognition of NBSI by the Clivia aptamer (Extended Data Fig. 2b). Additionally, one Mg^2+^ ion (M1) was observed in vicinity of the binding pocket, with one hydrated water W_M1_ forming two hydrogen bonds with the 2′-OH group of U8 and the O6 atom of G28, aiding in the formation of the binding pocket (Extended Data Fig. 2c).

### Investigation of RNA folding and recognition of NBSI

**Fig. 3:**
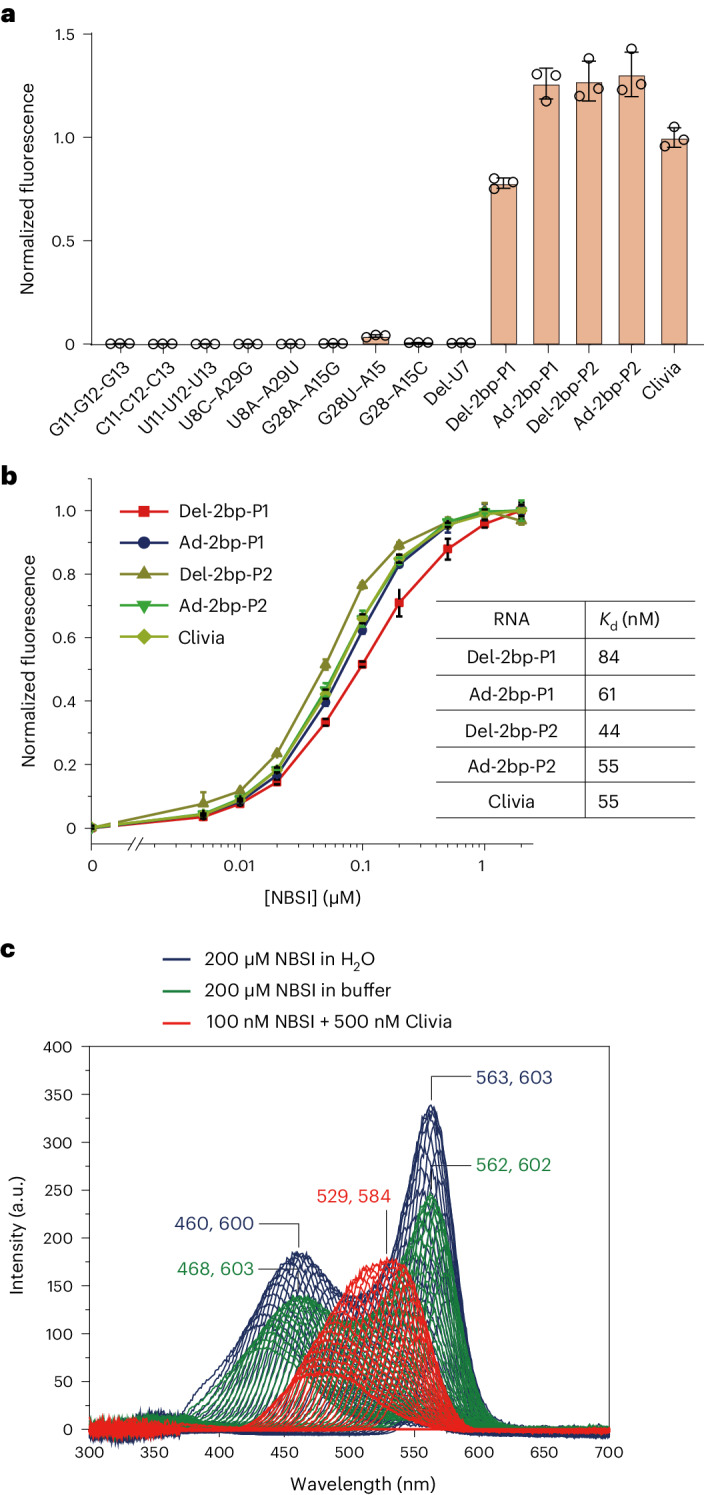
Fluorescence assay of the Clivia aptamer and mutants. **a**,**b**, Fluorescence assay of the Clivia aptamer (**a**) and structure-guided mutants (**b**). Here, Del-2bp-P1 indicates that G2-A3 and U34-C35 were removed from stem P1, Ad-2bp-P1 indicates that G(−1)-G0 and C37-C38 were added to stem P1, Del-2bp-P2 indicates that U16-G17 and C26-A27 were removed from stem P2 and Ad-2bp-P2 indicates that C-C and G-G were added to the apical end of stem P2. The activated fluorescence of NBSI by Clivia mutants from three independently repeated experiments is normalized for comparison with the WT Clivia aptamer. Data represent the mean ± s.d. from three replicates. **c**, The 3D fluorescence assay of fluorophore NBSI in the presence of H_2_O (blue), buffer (green) or Clivia (red). In the presence of water or buffer, free NBSI exhibits two excitation and emission wavelength peaks at approximately (460 nm, 600 nm) and (560 nm, 600 nm), while Clivia-bound NBSI exhibits single excitation (529 nm) and emission (584 nm) wavelength peaks. Source data

**Extended Data Fig. 3:**
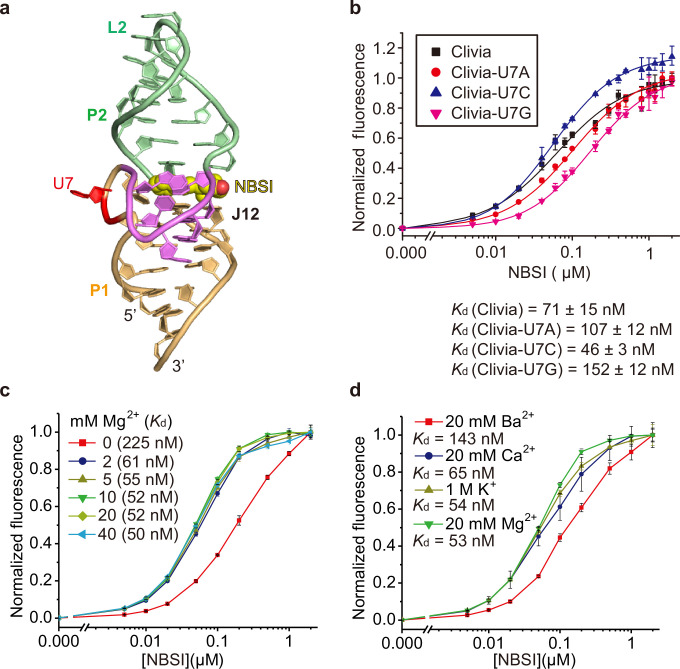
Fluorescence assay of U7 mutants and metal dependence test of Clivia aptamer. (**a**) The tertiary structure of Clivia aptamer with the protruded U7 residue in red. (**b**) The fluorescence assay of the substitution U7 with A, G and C shows that all the substitutions are tolerated. The activated fluorescence of NBSI by Clivia mutants U7A, U7G and U7C from three independently-repeated experiments are normalized for comparison with the wild-type (WT) Clivia aptamer. Data represent the mean ± s.d. from three replicates. (**c, d**) Fluorescence assay of Clivia aptamer binding with NBSI in the presence of different concentration of Mg^2+^ (**c**) and varied cations (**d**). All the titrations are performed three times independently. Data represent the mean ± s.d. from three replicates. Source data

On the basis of the architecture of the Clivia–NBSI complex, we introduced residue-specific substitutions to detect their contributions to RNA folding and NBSI recognition (Fig. 3a). As shown in Fig. 1e–g, A11-A12-A13 formed three consecutive stacked base triples below the binding pocket. Substitutions of A11-A12-A13 to G11-G12-G13, C11-C12-C13 and U11-U12-U13 all disrupted binding activity (Fig. 3a). Replacement of U8–A29, located below the bound NBSI, with either U8C–A29G or U8A–A29U resulted in a complete loss of binding activity (Fig. 3a). Then, we substituted the noncanonical base pair G28–A15, located above the bound NBSI. The binding activity of G28A–A15G and G28–A15C was completely abolished, while G28U–A15 resulted in severely reduced binding activity (Fig. 3a). It was noted that U7 protrudes from the main helical architecture of the Clivia monomer (Extended Data Fig. 3a) and participates in the molecular interaction of mol A and mol B in the asymmetry unit by partially stacking on the variable loop of stem P2 (Supplementary Fig. 1b). The deletion of U7 (Del-U7) resulted in a complete loss of NBSI-binding activity (Fig. 3a) but the substitution of U7 with C, G or A was tolerated (Extended Data Fig. 3b). As mentioned before, the NBSI-binding pocket is sandwiched between the coaxially stacked stems P1 and P2 in the tertiary fold. To assess the effect of stem length on NBSI-binding activity, stem P1 and stem P2 were elongated and shortened by two base pairs, respectively. Normalized fluorescence experiments showed that the binding activity and binding affinity of these mutants were comparable to those of the wild-type (WT) construct (Fig. 3a,b).

As shown in Fig. 1d and Supplementary Fig. 2, two bound Mg^2+^ cations (M1 and M2) were observed adjacent to the NBSI-binding pocket. To evaluate the contributions of the cations to the structural folding of Clivia and the ligand recognition of NBSI, we tested the binding activities of Clivia with NBSI in the existence of different concentrations of Mg^2+^ (Extended Data Fig. 3c) and other cations, including Ca^2+^, K^+^ and Ba^2+^ (Extended Data Fig. 3d). In the absence of Mg^2+^, the binding affinity of Clivia and NBSI was 225 nM (Extended Data Fig. 3c). In the presence of Mg^2+^ with concentration varied from 2 to 40 mM, the binding affinity became 50–61 nM (Extended Data Fig. 3c). The binding activity was not affected when the cations were changed to 20 mM Ca^2+^ or 1 M K^+^. However, when replaced with 20 mM Ba^2+^, the binding affinity was decreased to 143 nM (Extended Data Fig. 3d).

**Extended Data Fig. 4:**
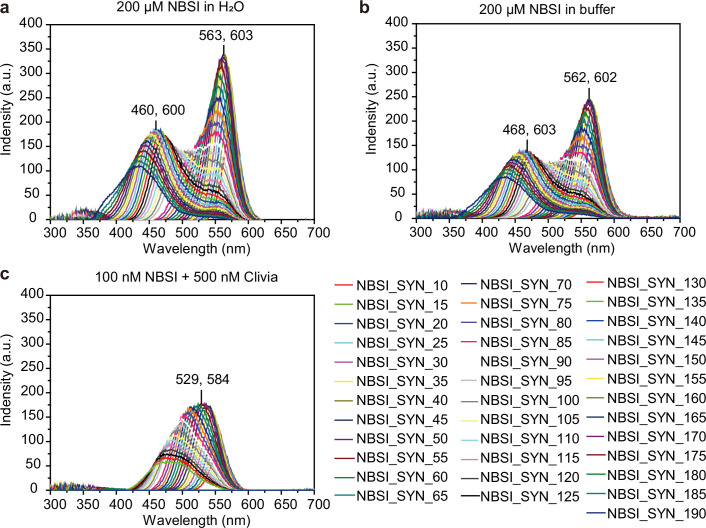
3D fluorescence assay of free NBSI and Clivia-bound NBSI. (**a**) 3D fluorescence spectrometry of NBSI in water, the maximum excitation and emission wavelengths are 563 nm and 603 nm, respectively. (**b**) 3D fluorescence spectrometry of NBSI in buffer. The maximum excitation and emission wavelengths are 562 nm and 602 nm, respectively. (**c**) 3D fluorescence spectrometry of NBSI-Clivia complex in buffer. The maximum excitation and emission wavelengths are 529 nm and 584 nm, respectively. In 3D fluorescence assay, the initial difference ‘Delta start’ between emission wavelength and excitation wavelength was 10 nm, the increment of wavelength difference ‘Delta increment’ was 5 nm, and the maximum wavelength difference ‘Delta stop’ was 190 nm. Source data

In addition to detecting the structural folding features of Clivia, we investigated the fluorescence characteristics of NBSI under different conditions, including in water, in buffer and in the presence of Clivia. As shown in the three-dimensional (3D) fluorescence spectra (Fig. 3c and Extended Data Fig. 4), NBSI exhibited two distinct excitation peaks at approximately 460 and 560 nm in both water and buffer environments. Interestingly, upon the introduction of Clivia, the excitation spectrum of NBSI showed a single peak centered around 530 nm. Moreover, the comparison of the emission intensity of Clivia-bound NBSI (100 nM) to that of free NBSI at the same concentration revealed that the emission of free NBSI was negligible in comparison (Fig. 3c and Extended Data Fig. 4). These observations collectively suggest that the interaction between NBSI and Clivia induces a notable alteration in the intricate fluorescence attributes of NBSI.

### Photophysical properties of Clivia-bound NBSI derivatives

Our previous findings demonstrated that modifying the fluorophore structure can effectively alter its photophysical properties, resulting in a broader range of fluorescence wavelengths, including a larger Stokes shift^19,22^. As shown in the binding pocket of NBSI (Fig. 2a), the terminal (2-hydroxyethyl)(methyl)amino group of NBSI points outward from the ligand-binding pocket of the Clivia aptamer, providing ample space for NBSI modification.

**Fig. 4:**
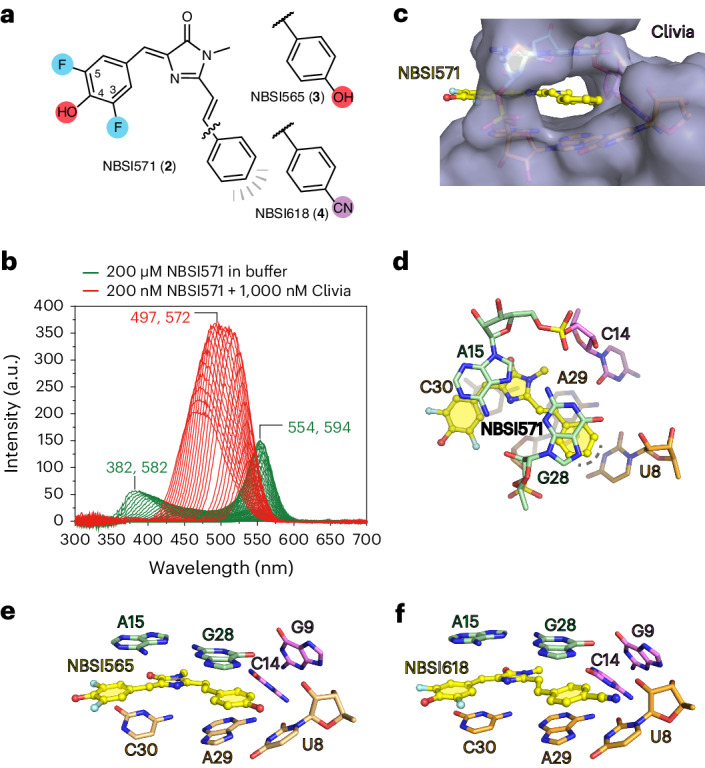
Structure of the Clivia aptamer binding with modified NBSI analogs. **a**, The chemical structure of NBSI analogs NBSI571, NBSI565 and NBSI618. **b**, The 3D fluorescence assay of free (green) and Clivia-bound (red) NBSI571. In the presence of buffer, free NBSI571 exhibits two excitation and emission wavelength peaks at (382 nm, 582 nm) and (554 nm, 594 nm), while Clivia-bound NBSI571 exhibits single excitation (497 nm) and emission (572 nm) wavelength peaks. **c**, Surface representation of the Clivia aptamer bound to NBSI571 shown in stick representation. **d**, Binding pocket composition of NBSI571. The binding pocket of NBSI571 in the Clivia aptamer suggests that there is room for additional modifications on the phenyl moiety of NBSI571 to enhance dye properties. **e**,**f**, Binding pocket composition of NBSI565 (**e**) and NBSI618 (**f**). Source data

**Extended Data Fig. 5:**
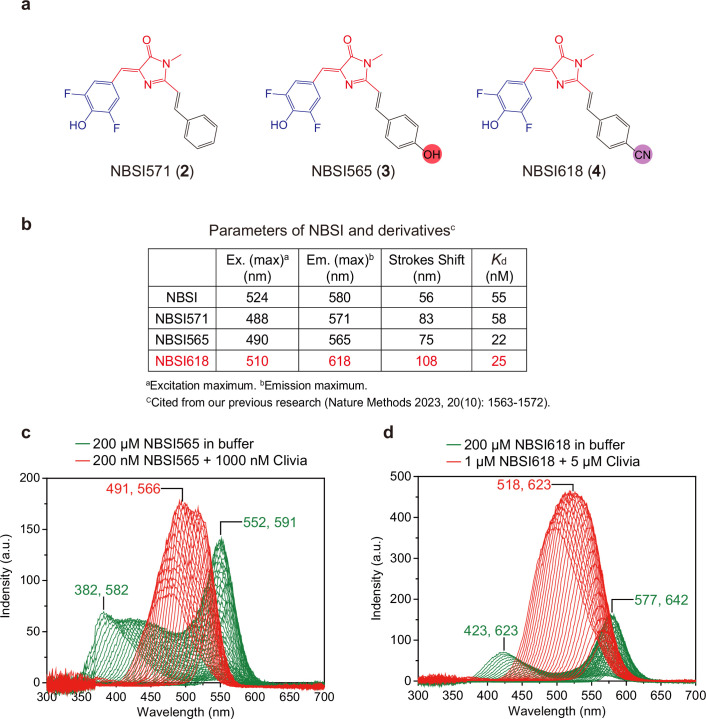
3D fluorescence assay of fluorophore NBSI565 and NBSI618. (**a**) Chemical structure of NBSI571, NBSI565 and NBSI618. (**b**) The photophysical parameters of NBSI and derivatives, the data was cited for our previous research (Nature Methods 20(10): 1563-1572). (**c**) 3D fluorescence assay of fluorophore NBSI565 in the presence of buffer (green) or Clivia (red). In the presence of buffer, Free NBSI565 exhibits two excitation and emission wavelengths peaks at approximately (382 nm, 582 nM) and (552 nm, 591 nm), while Clivia-bound NBSI565 exhibits a single excitation and emission wavelengths peak at (491 nm, 566 nm). (**d**) 3D fluorescence assay of fluorophore NBSI618 in the presence of buffer (green) or Clivia (red). In the presence of buffer, Free NBSI618 exhibits two excitation and emission wavelengths peaks at approximately (423 nm, 623 nm) and (577 nm, 642 nm), while Clivia-bound NBSI618 exhibits a single excitation peak at (518 nm, 623 nm). Source data

In the reported NBSI derivative, NBSI571 (**2**)^22^, this terminal (2-hydroxyethyl)(methyl)amino group is replaced by a minor hydroxyl group at position 4, along with two additional fluoride atoms at adjacent positions 3 and 5 of the benzylidene moiety (Fig. 4a). The fluorescence binding experiments revealed that the binding affinity of NBSI571 (*K*_d_ = 58 nM) remained unaffected by the substitutions compared to NBSI (*K*_d_ = 55 nM) (Extended Data Fig. 5a,b)^22^. Next, 3D fluorescence spectra were generated for both free NBSI571 in buffer and Clivia-bound NBSI571 (Fig. 4b). Free NBSI571 in buffer exhibited two excitation wavelength peaks at 382 and 554 nm, whereas Clivia-bound NBSI571 displayed only one excitation wavelength peak at 497 nm. The binding of Clivia resulted in a notable alteration in the fluorescence characteristics of NBSI571. Then, we performed cocrystallization of the Clivia aptamer with NBSI571. The binding pocket of the Clivia–NBSI571 complex structure revealed that the three newly attached groups (3,5-F and 4-OH) of NBSI571 project outward in a manner similar to the (2-hydroxyethyl)(methyl)amino group of NBSI (Figs. 2a,b and 4c,d).

Two additional reported derivatives of NBSI, designated NBSI565 (**3**) and NBSI618 (**4**), were generated by introducing a 4-OH or 4-CN group to the styryl moiety of NBSI571, respectively (Extended Data Fig. 5a)^22^. NBSI565 had an excitation maximum at 490 nm and an emission maximum at 565 nm, while its binding affinity was *K*_d_ = 22 nM (Extended Data Fig. 5b)^22^. Our 3D fluorescence spectra of free NBSI565 in buffer and Clivia-bound NBSI565 revealed that free NBSI565 in buffer exhibited two excitation wavelength peaks at 382 and 552 nm, whereas Clivia-bound NBSI565 displayed only one excitation wavelength peak at 491 nm (Extended Data Fig. 5c). Structure determination of the Clivia–NBSI565 complex demonstrated a remarkable resemblance of the binding pocket architecture to that of NBSI571 and NBSI. NBSI565 was stacked in the binding pocket with the attached 4-OH group residing at the terminus of the binding pocket (Fig. 4e).

In NBSI618, a 4-CN group was incorporated into the styryl moiety and resulted in an excitation maximum at 510 nm and an emission maximum at 618 nm, thereby achieving a substantial Stokes shift of 108 nm (Extended Data Fig. 5a,b)^22^. The distinct 3D fluorescence spectra of free NBSI618 in buffer and Clivia-bound NBSI618 clearly demonstrated that the binding of Clivia induced a notable change in the fluorescence properties of NBSI618, as evidenced by a shift in the excitation wavelength (Extended Data Fig. 5d). Structure determination of the Clivia–NBSI618 complex revealed that NBSI618 retained the ligand conformation and interaction observed for NBSI (Fig. 2), NBSI571 (Fig. 4c,d) and NBSI565 (Fig. 4e). The elongated 4-cyano-benzylidene moiety of NBSI618 was capsuled within the binding pocket of Clivia (Fig. 4f). Detailed analysis revealed a substantial increase in the buried area of the fluorophore within Clivia, expanding from 383.2 Å^2^ for NBSI571 to 412.1 Å^2^ for NBSI618. This enhancement highlights the robust ligand-binding pocket of the Clivia aptamer, potentially contributing to the remarkably large Stokes shift observed in NBSI derivatives. It is worth noting that the largest Stokes shift observed for NBSI618 binding to Clivia may also partially stem from the intrinsic molecular properties of NBSI618.

### Orthogonal application of Clivia and alternate aptamers

**Fig. 5:**
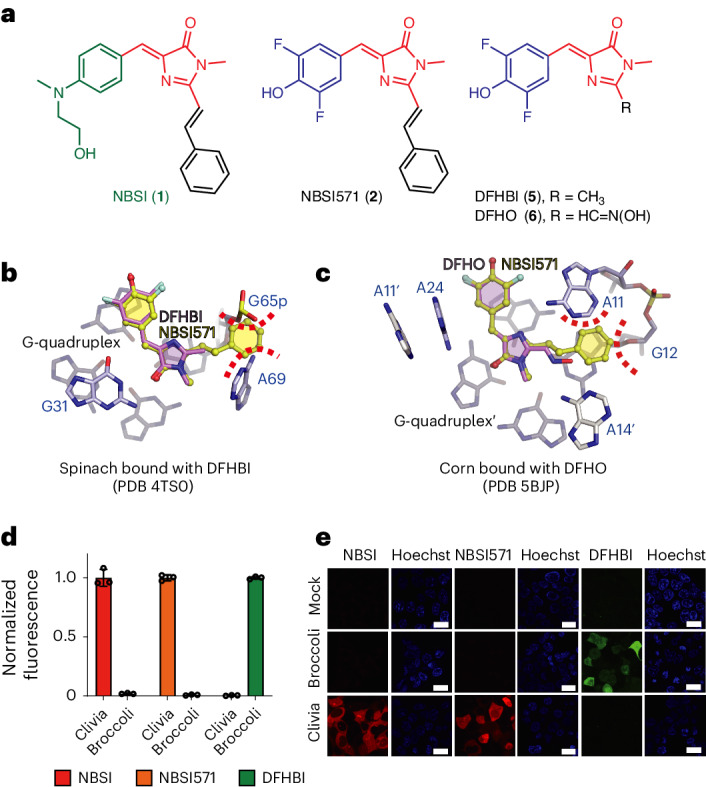
Structural basis underlying the orthogonal use of Clivia and alternate aptamers. **a**, Chemical structure comparison of NBSI, DFHBI, DFHO and NBSI571. **b**, Docking of NBSI571 into the binding pocket of Spinach bound to DFHBI (PDB 4TS0). **c**, Docking of NBSI571 into the binding pocket of Corn fluorogenic aptamer bound to DFHO (PDB 5BJP) results in a steric clash of the phenyl moiety of NBSI571 with the base of A11 and the sugar of G12 from Corn. **d**, In vitro fluorescence assay of the Clivia and Broccoli aptamers binding to NBSI, NBSI571 and DFHBI. The in vitro fluorescence activation of NBSI, NBSI571 and DFHBI by the Broccoli aptamer is normalized for comparison with the Clivia aptamer. All tests were performed three times independently. Data represent the mean ± s.d. from three replicates. **e**, Activation of NBSI, NBSI571 and DFHBI by the Clivia and Broccoli aptamers in HEK293T cells. Scale bars, 20 μm. Source data

The chemical structure of NBSI consists of an electron donor, an electron acceptor and a bridged *π* conjugate structure (Fig. 5a). We found that the bridged *π* conjugate substructure present in NBSI and its derivatives also exists in the well-known conventional fluorophores DFHBI (**5**) and DFHO (**6**) (Fig. 5a), which are capable of being activated by various RNA aptamers, including Spinach^24^, iSpinach^25^, Corn^11^ and Broccoli^26^. In addition, NBSI571 contains a hydroxyl group at position 4 and two fluoride atoms at adjacent positions 3 and 5 of the benzylidene moiety present in DFHBI and DFHO.

**Extended Data Fig. 6:**
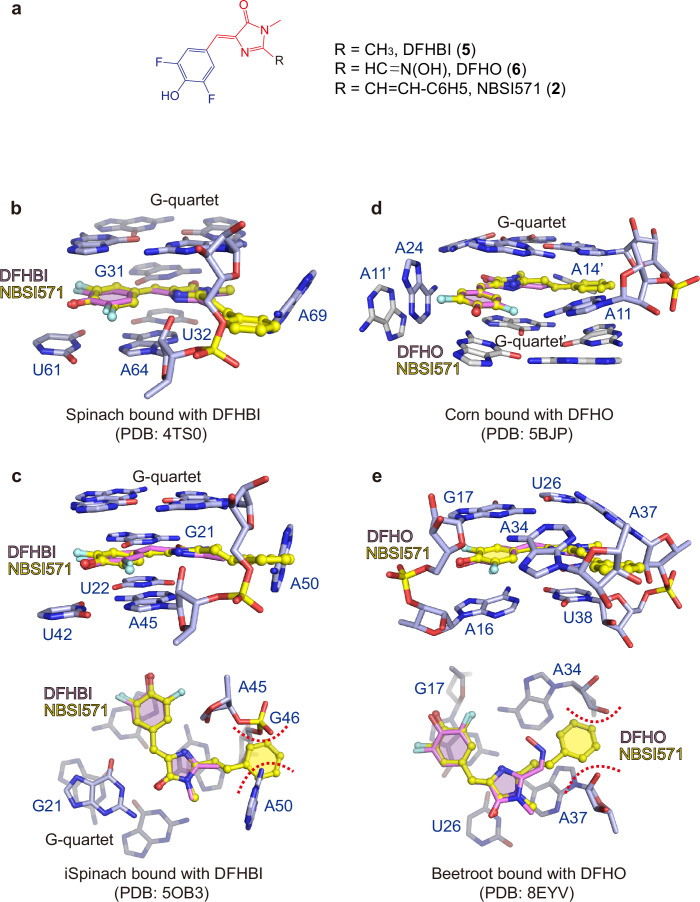
Docking of NBSI571 into the DFHBI or DFHO binding pocket of other fluorogenic RNA aptamer. (**a**) Chemical structure of DFHBI, DFHO and NBSI571. (**b**) Docking of NBSI571 into the binding pocket of Spinach fluorogenic aptamer bound with DFHBI (PDB: 4TS0). (**c**) Docking of NBSI571 into the binding pocket of iSpinach fluorogenic aptamer bound with DFHBI (PDB: 5OB3) results in steric clash between the phenyl moiety of NBSI571, and the phosphate of G46 and the base of A50 from iSpinach. (**d**) Docking of NBSI571 into the binding pocket of Corn fluorogenic aptamer bound with DFHO (PDB: 5BJP). (**e**) Docking of NBSI571 into the binding pocket of Beetroot fluorogenic aptamer bound with DFHO (PDB: 8EYV) results in steric clash between the phenyl moiety of NBSI571, and the sugars of A34 and A37 from Beetroot.

Given the strong structural resemblance of NBSI571 with DFHBI and DFHO (highlighted in blue and red, respectively, in Fig. 5a), we conducted a detailed comparison of the binding patterns of these fluorophores with their corresponding RNA-based fluorogenic aptamers (Fig. 5b,c and Extended Data Fig. 6). It was observed that both DFHBI and DFHO are recognized by the same molecular moiety, including the benzylidene substructure (shown in bule) and the bridged *π* conjugate substructure (shown in red), present in NBSI571 (Fig. 5a and Extended Data Fig. 6a). However, our attempts to dock NBSI571 into the binding pockets of Spinach (Protein Data Bank (PDB) 4TS0), iSpinach (PDB 5OB3), Corn (PDB 5BJP) and Beetroot (PDB 8EYV) revealed severe steric clashes with the phenyl group of NBSI571 (Fig. 5b,c and Extended Data Fig. 6). These results indicate the unique fluorophore recognition pattern of Clivia, implying that NBSI and its derivatives, including NBSI571, can be used in an orthogonal manner for the simultaneous tracking of diverse RNA molecules alongside other fluorophores such as DFHBI and DFHO.

**Extended Data Fig. 7:**
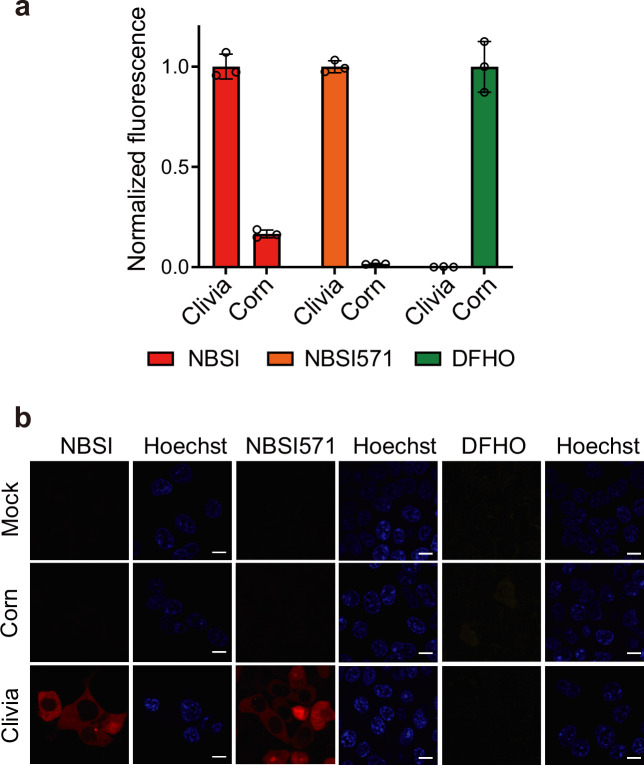
The biorthogonality of Clivia with Corn. (**a**) In vitro fluorescence assay of Clivia and Corn aptamer binding with NBSI, NBSI571 and DFHO, respectively. The in vitro fluorescence activation of NBSI, NBSI571 and DFHO by Corn aptamer are normalized for comparison with the Clivia aptamer. Three independent experiments were carried out with similar results. Data represent the mean ± s.d. from three replicates. (**b**) Activation of NBSI, NBSI571 and DFHO by Clivia and Corn aptamer in HEK293T cells. Scale bar, 10 μm. Source data

To explore the potential orthogonal use of Clivia and alternate aptamers, we conducted an in vitro fluorescence assay of the Clivia and Broccoli aptamers binding to NBSI, NBSI571 and DFHBI (Fig. 5d). The results revealed that Clivia exclusively triggered the fluorescence of NBSI or NBSI571 in the presence of DFHBI, while Broccoli activated the fluorescence of only DFHBI (Fig. 5d). In live-cell imaging, after HEK293T cells were transfected with plasmids encoding either Clivia or Broccoli, distinct fluorescence responses were observed. When these cells were subjected to NBSI and NBSI571, the cells encoding Clivia emitted a vibrant red fluorescence. Conversely, when treated with DFHBI, the cells encoding the Broccoli aptamer displayed a vivid green fluorescence (Fig. 5e). Additionally, we performed an in vitro fluorescence assay of the Clivia and Corn aptamers binding to NBSI, NBSI571 and DFHO (Extended Data Fig. 7a). We observed that Clivia did not activate the fluorescence of DFHO, which was further supported by live-cell imaging experiments in HEK293T cells (Extended Data Fig. 7b). This stark contrast in fluorescence colors serves as a clear indicator of the specific interactions between these aptamers and their respective fluorophores, thus confirming Clivia’s unique ability to distinguish and activate fluorescence in response to NBSI and its derivatives.

### Design of multivalent Clivia fluorogenic aptamers

Accurate spatiotemporal tracking of RNA molecules, particularly those that are present in low abundance, relies on sensitive detection and quantification. Furthermore, high concentrations of fluorophores may potentially interfere with normal cellular activities. To enhance the imaging sensitivity of individual RNA molecules in the presence of a controlled concentration of fluorophore NBSI, we embarked on the design of constructs containing multiple NBSI-binding pockets arranged in tandem within a single sequence.

**Fig. 6:**
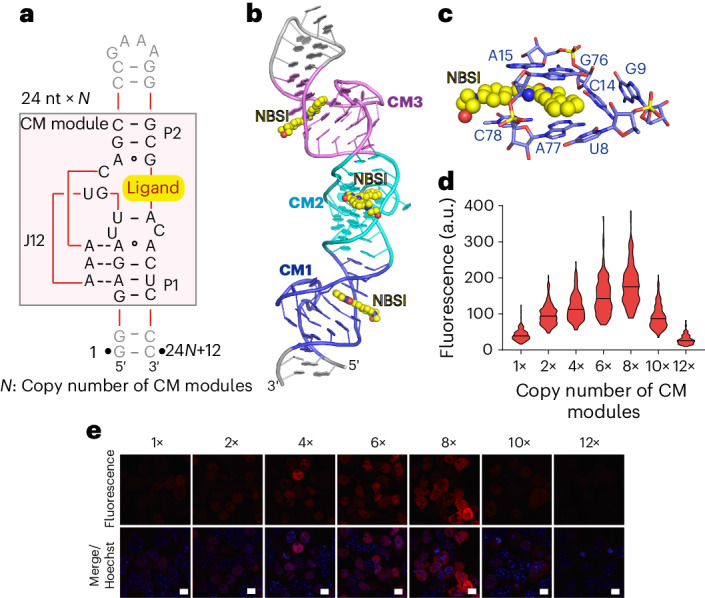
Design, fluorescence assay and structure of NBSI-binding modules in tandem arrays. **a**, Design of multivalent Clivia fluorogenic aptamers containing multiple NBSI-binding modules (CM modules) in a tandem array. The linked stem was reduced to 5 bp (3 bp from stem P1 and 2 bp from stem P2). Each NBSI-binding module contained 24 nucleotides. **b**, Tertiary structure of Clivia_III with three NBSI-binding modules in a tandem array stacking continuously in a long helix. **c**, Composition of the first NBSI-binding pocket in Clivia_III. **d**,**e**, Live-cell imaging (**e**) and quantitation of the cellular fluorescence (**d**) for the multivalent Clivia fluorogenic aptamers containing 1–12 NBSI-binding modules. Scale bars, 10 μm. Data represent the mean ± s.d. (*n* = 150 cells). Source data

Inspired by the coaxial tertiary architecture of the monomeric Clivia aptamer and the substitution results from Figs. 1d and 3a,b showing that the alteration of stem length had a minor effect on the fluorescence activation of Clivia, we used multivalency-based strategies^27^ to design a multivalent Clivia fluorogenic aptamer that houses repeated NBSI-binding modules from Clivia (CM modules) in a coaxial fold, termed Clivia_*N* (where *N* represents the copy number of CM modules) (Fig. 6a). Guided by the tertiary structure folding of monomeric Clivia (Fig. 1c,d), we used 3 bp from the north terminus of stem P1 and 2 bp from the south terminus of stem P2 as the linked stem (Fig. 6a). Therefore, the size of each NBSI-binding module in the multivalent Clivia fluorogenic aptamer was decreased from more than 36 nucleotides to 24 nucleotides (Figs. 1c and 6a).

**Extended Data Fig. 8:**
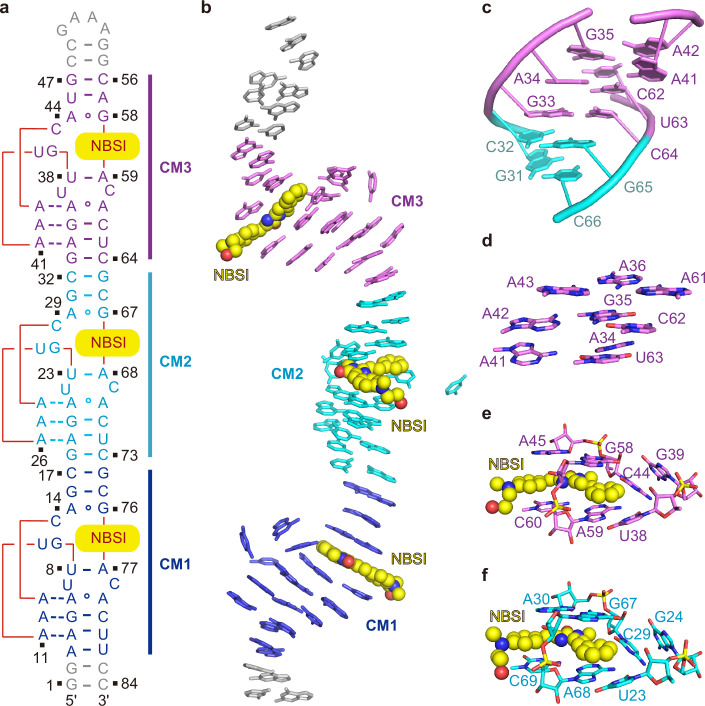
Tertiary structure of Clivia_III-NBSI. (**a**) The tertiary structure of Clivia_III-NBSI was shown schematically. The three NBSI binding modules were shown in blue (CM1), cyan (CM2) and magenta (CM3) from the south to north respectively. (**b**) Continuous stacking helical architecture was formed by the pairing residues and three bound NBSI molecules. (**c**) The folding of the connected stem between CM2 and CM3. A42 and A41 form a-minor interaction with the connected stem. (**d**) A-minor base triple interaction in module CM3 is shown and all the involved residues are represented in stick. (**e-f**) Two NBSI binding pockets CM2 and CM3 are shown in stick respectively. Each of them folds independently with similar conformations in Clivia_III-NBSI structure.

**Extended Data Fig. 9:**
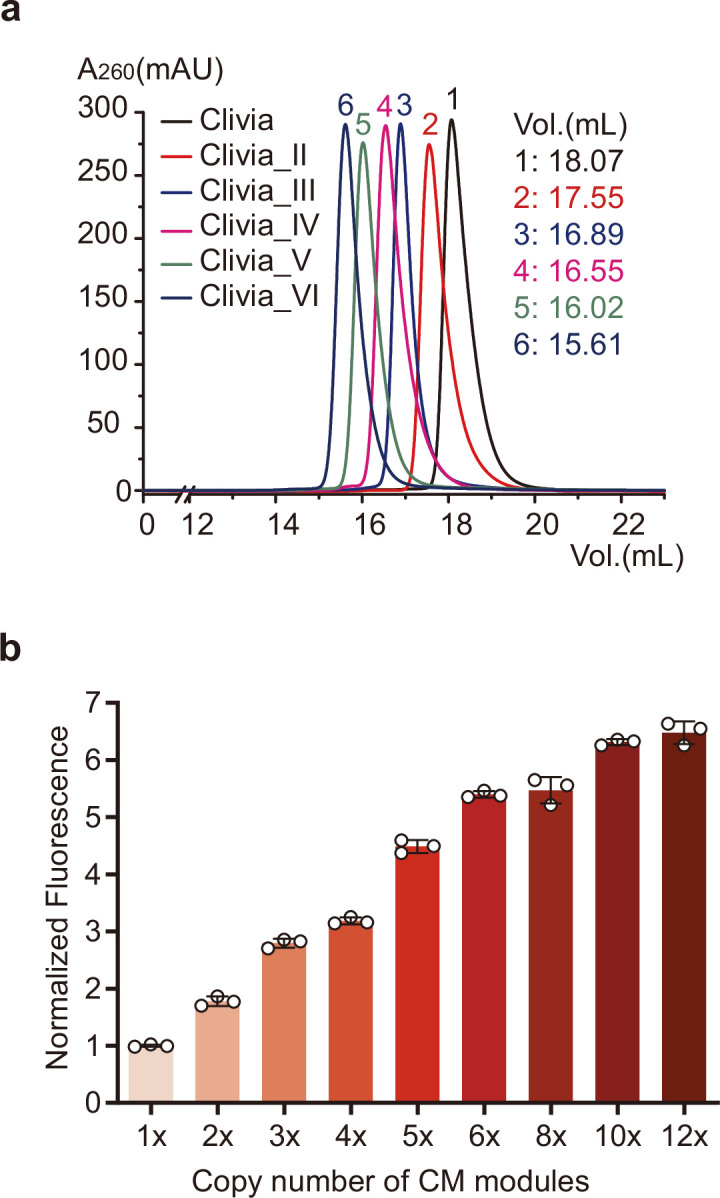
Gel-filtration analysis and in vitro fluorescence assay of multivalent Clivia fluorogenic aptamer. (**a**) The solution state of multivalent Clivia fluorogenic aptamer containing varying number of NBSI binding modules from 1 to 6 were tested by size-exclusion experiment with SuperoseTM Increase 10/300 GL column. The running buffer contains 50 mM HEPES, pH 6.8, 50 mM NaCl and 5 mM MgCl_2_. All the RNA molecules exist homogenously as monomers in solution. (**b**) In vitro fluorescence assay of multivalent Clivia fluorogenic aptamer reveal increased fluorescence intensity with an increasing number of NBSI binding modules, all in the presence of the same concentration of NBSI. The activated fluorescence of NBSI by multivalent Clivia fluorogenic aptamer from three independently repeated experiments are normalized for comparison with the single WT Clivia aptamer. Data represent the mean ± s.d. from three replicates. Source data

To detect whether the repeated NBSI-binding pockets were affected by the tandem-arrayed pattern, we set out to solve the complex structure of the multivalent Clivia fluorogenic aptamer bound to NBSI and succeeded with the Clivia_III construct containing three CM modules (Fig. 6b and Extended Data Fig. 8). As shown in Fig. 6b and Extended Data Fig. 8a,b, the three NBSI-binding modules, CM1, CM2 and CM3, stacked continuously with the intercalated linked stem in the Clivia_III–NBSI complex structure. The 5-bp linked stem between every two NBSI-binding modules was involved in A-minor interactions (A42 and A41) with the NBSI-binding pocket situated above it (Fig. 6c and Extended Data Fig. 8a–d), as shown in the monomer structure (Fig. 1c,d). The residues lining each binding pocket were not affected by the tandem array of the NBSI-binding modules (Fig. 6b and Extended Data Fig. 8e,f). Then, we used size-exclusion chromatography to examine the solution state of the multivalent Clivia fluorogenic aptamers containing 1–6 repeated CM modules. Our analysis revealed that all aptamers maintained a homogeneous monomeric state in solution (Extended Data Fig. 9a). These observations establish the structural basis for the efficient application of multivalent Clivia fluorogenic aptamers for RNA localization and trafficking.

To assess the practical utility of multivalent Clivia fluorogenic aptamers, we conducted in vitro fluorescence assays using constructs with 1–12 repeated CM modules. The results exhibited enhanced fluorophore sensitivity and linearly increasing intensity with the growing number of CM modules in the same condition (Extended Data Fig. 9b). Furthermore, we transfected plasmids encoding multivalent Clivia fluorogenic aptamers with 1–12 CM modules into HEK293T cells. Upon incubation with the same concentration of NBSI, the intracellular fluorescence of the multivalent Clivia–NBSI complex exhibited a notable and consistent increase when up to eight NBSI-binding modules were arranged in tandem (Fig. 6d,e). However, intriguingly, when the copy number exceeded this point (10–12 CM modules), there was a decrease in fluorescence intensity. Therefore, we concluded that the optimal copy number of CM modules in the multivalent Clivia fluorogenic aptamer for maximal fluorescence enhancement is 8 (Fig. 6d,e).

### Importance and comparison of base triples in RNA structures

**Extended Data Fig. 10:**
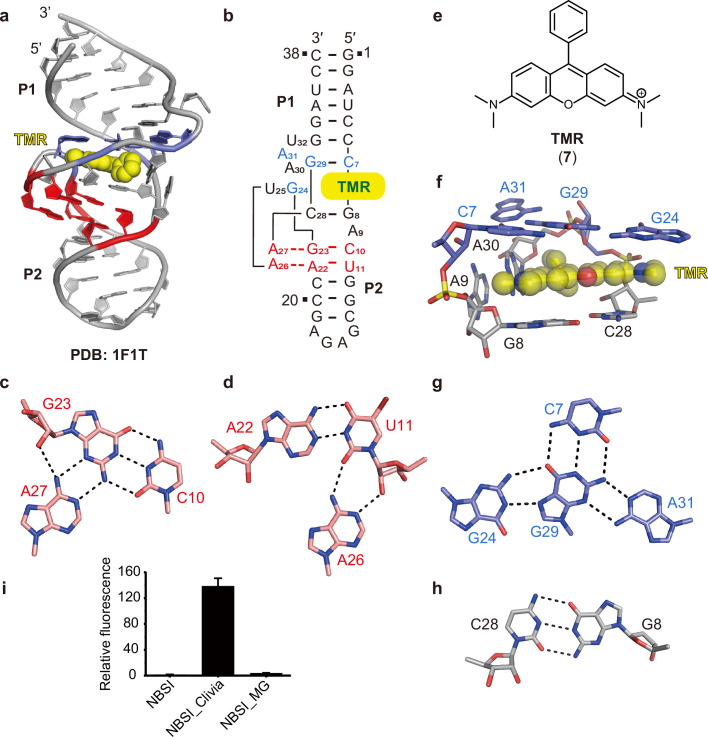
Structure of MG aptamer bound with TMR. (**a-b**) The tertiary structure of MG aptamer bound with TMR (PDB:1F1T) is shown in cartoon (**a**) and schematic (**b**). The RNA folds in helical architecture with TMR intercalates in the center. (**c-d**) Two A-minor base triples forming in the junction of MG aptamer are shown in stick. (**e**) Chemical structure of TMR. (**f-h**) Residues interaction lining TMR binding pocket of MG aptamer are shown in stick. One base tetrad (**g**) and one base pair G8-C28 (**f**) stack on both sides of the bound TMR. (**i**) In vitro fluorescence assay of NBSI binding with Clivia and MG aptamer, respectively. MG aptamer cannot activate the fluorescence of NBSI. Three experiments were carried out independently. Data represent the mean ± s.d. from three replicates. Source data

As shown in Fig. 1, there are four non-G-quadplex tiers that constitute the platform below the bound ligand in the Clivia–NBSI complex structure, which are all composed of base triples. The top tier is composed of a noncanonical base triple formed by three residues (U8, A29 and C30) from junction J12 (Fig. 2e). Three additional tiers are formed by base triples involving three consecutive adenines A11, A12 and A13 from the junction region (Fig. 1e–g and Extended Data Fig. 1). Compared with other solved RNA fluorogenic molecule structures, we found that base triples also constitute the foundational tiers within the tertiary complex structure of the MG aptamer bound to TMR (**7**) dye (PDB 1F1T; Extended Data Fig. 10). As shown in Fig. 1d and Extended Data Fig. 10a,b, both the MG aptamer and the Clivia aptamer feature a helical scaffold with the fluorogenic dye binding at the center of the structure. Two base triples formed by A26 and A27 from the junction region and the minor groove of two base pairs (A22–U11 and G23–C10) from stem P2 in the MG aptamer show identical interactions to base triples featuring A11 and A12 in the Clivia aptamer (Fig. 1e–g and Extended Data Fig. 10c,d). Despite the high similarity in the overall helical structure and the platform of base triples, it is important to note that the specific recognition module and the bound fluorophore molecule structures are completely distinct (Fig. 2 and Extended Data Fig. 10e,h). A fluorescence assay of the MG aptamer in the presence of NBSI indicated that the MG aptamer could not activate the fluorescence of NBSI, contrasting with the activation behavior observed for Clivia (Extended Data Fig. 10i). This highlights the inherent versatility of RNA molecules and their specificity in recognizing small fluorophore molecules with diverse chemical structures. Additionally, base triples were identified as components of the drug-binding pocket in a riboswitch-like RNA structure from the internal ribosome entry site of hepatitis C virus (PDB 3TZR; Supplementary Fig. 3a,b). The ligand SS0 (**8**) was recognized by the G52–C111–A57 and U59–A109–A53 base triples (Supplementary Fig. 3c–e). This suggests that base triples may serve as common structural elements for interacting with small molecules.

## Discussion

Fluorescence microscopy has a vital role in numerous fundamental studies including biological macromolecule interactions, biological macromolecule trafficking and clinical applications. RNA and DNA mimics of fluorescence proteins have recently been developed to track the nucleic acids involved in cellular activity^9–12,14,16–19,24,28^. However, most in vitro selected fluorescent aptamers adopt a G-quadruplex scaffold, the use of which is limited by the consideration of some G-quadruplex-specific proteins^15^. Our structural investigation of the Clivia aptamer bound to NBSI or NBSI derivatives revealed that the Clivia aptamer adopts a non-G-quadruplex scaffold supporting a specific ligand-binding pocket, suggesting a new fluorescence activation mechanism.

Different RNA molecules perform various functions within the cell. Concurrently monitoring these distinct RNA types is pivotal for comprehending cellular dynamics, uncovering disease mechanisms and deciphering the intricate regulatory networks that govern biology. Specific 3D structures enable Clivia to selectively interact with NBSI and NBSI derivatives. Aptamers such as Broccoli have distinct binding pockets and molecular arrangements that favor their interaction with fluorophores such as DFHBI, leading to fluorescence activation. Unique binding pockets and molecular recognition patterns establish the structural basis for the orthogonal use of Clivia and other aptamers. The in vitro and live-cell imaging results herein provide compelling and conclusive evidence supporting the orthogonal utility of NBSI, NBSI571 and DFHBI. In other words, they affirm that these three fluorophores can be effectively used in a mutually exclusive manner for various RNA-tracking applications, each responding selectively to its corresponding aptamer without interference from the others. This capacity for orthogonal usage holds great promise for multifaceted RNA imaging and research endeavors, which has the potential to greatly advance our understanding of the intricate biological processes involving RNA molecules.

The ideal properties of fluorescence toolkits include high photostability, elevated fluorescence quantum yields, a great molar extinction coefficient and a large Stokes shift (>100 nm)^29^. Large Stokes shifts constitute a wide gap between the excitation and emission maxima, which diminishes self-absorption of the emitted light^20,29,30^ and offers the possibility to apply multicolor imaging at a single wavelength^20,29,30^. Through rational structure-based modification, the newly identified Clivia aptamer and its corresponding NBSI derivative generated a notably large Stokes shift in the red spectrum up to 108 nm. Its photophysical properties overscore most known fluorogenic RNA molecules, including the Spinach^9,10,24^, Mango^12,14^, Corn^11^, DIR2 (refs. ^16,17^) and Pepper^18,19^ aptamers, which show only a modest difference between their excitation and emission maxima. Our complex structures of Clivia bound to NBSI derivatives further confirm that the ligand-binding pockets of Clivia allow optimization or modifications of the cognate fluorophore, establishing the thorough structural basis for creating NBSI scaffold-based fluorophores with a more favorable red-shift wavelength and for further engineering of the Clivia aptamer.

A tandem-arrayed pattern is often used in certain RNA fluorogenic molecules, leading to a considerable enhancement of the fluorescence signal intensity^31,32^. However, as the arrayed number of independent fluorescence aptamers increases, the folding efficiency decreases because of cross-talk and mispairing problems between neighboring aptamers^31^. This issue calls for compact fluorescent RNA modules exhibiting robust tertiary folding, which would effectively enhance the fluorescence intensity of tandem array imaging. On the basis of the tertiary structure of the Clivia aptamer, we designed multivalent Clivia fluorogenic aptamers containing tandem-arrayed coaxial NBSI-binding modules within a single molecule (Fig. 6a). Remarkably, each NBSI-binding module in the multivalent Clivia fluorogenic aptamer only contains 24 nucleotides, which is smaller than most prevailing fluorogenic molecule-binding modules. Our in vitro experiment, coupled with structural research, revealed that these multivalent Clivia fluorogenic aptamers folded as homogeneous monomers in solution (Extended Data Fig. 9a) and each NBSI-binding module formed coaxially within the tertiary structure (Fig. 6 and Extended Data Fig. 8). Subsequent in vivo investigations unveiled that the introduction of these multivalent Clivia fluorogenic aptamers within one extended helical structure led to a proportional increase in fluorescence activation as the copy number of NBSI-binding modules increased to eight (Extended Data Fig. 9b and Fig. 6d). The design of this new tandem array pattern greatly increases the number of fluorophore-binding modules within a certain length of fluorogenic RNA molecule and avoids the potential problems between neighboring independent aptamers; this provides some inspiration for the future design of other tandem-arrayed multivalent RNA molecules. Additionally, the excellent photophysical properties of tandem-arrayed coaxial modules within Clivia substantially increased the fluorescence intensity and improved the sensitivity of the aptamer at specific concentrations of NBSI and its derivatives, opening up new possibilities for diverse applications of the Clivia aptamer.

## Methods

### RNA preparation for crystallography

The corresponding DNA sequence of the Clivia aptamer was cloned into a pUT7 vector followed by the sequence of the hammerhead ribozyme under the control of a T7 promoter. Then, the plasmid was transformed into DH5α cells and amplified to a large scale. The extracted plasmid was linearized with the *Hin*d III restriction endonuclease and used as a template for transcription of the Clivia aptamer.

All the RNA samples for crystallization were prepared by in vitro transcription with bacteriophage T7 RNA polymerase. To facilitate crystallization, the apical loop of stem P2 of Clivia was modified with different alternative loops such as the GAAA tetraloop. After transcription, the RNA sample of Clivia was separated by denaturing urea polyacrylamide gel electrophoresis (PAGE) and further purified by anion-exchange chromatography and ethanol precipitation.

Purified Clivia RNA samples were annealed at 65 °C for 5 min in a buffer containing 50 mM HEPES pH 7.4, 125 mM KCl and 5 mM MgCl_2_ and then incubated on ice for 30–60 min. Because of the low water solubility of the ligand NBSI and its analogs, the purified Clivia samples were diluted to a low concentration and then combined with the ligand solution. The RNA–ligand complexes were concentrated to approximately 0.5 mM before crystallization.

### Crystallization

Crystallization of the Clivia RNA samples was performed with the sitting-drop vapor diffusion method by mixing samples at a 1:1 ratio with the reservoir solution at 16 °C. Crystals of the Clivia RNA samples with a GAAA loop replacement in the terminus of stem P2 were grown in conditions of 0.05 M MgCl_2_, 0.1 M HEPES pH 7.5 and 30% polyethylene glycol monomethyl ether 550 over 7 days. Crystals of the tandem Clivia_III construct appeared in crystallization conditions of 0.2 M MgCl_2_ and 20% polyethylene glycol 3350 over 7 days. The crystals of Clivia_III–NBSI were cryoprotected using well solution supplemented with 15% glycerol before flash-freezing in liquid nitrogen. For anomalous data collection, crystals of the Clivia samples were soaked in the above crystallization conditions supplemented with 5 mM Ir(NH_3_)_6_^3+^ at 4 °C for 13 h or 50 mM MnCl_2_ at 4 °C for 5 h.

### X-ray data collection and refinement

X-ray diffraction data of the Clivia aptamer were collected at 100 K from beamlines BL17U1, BL017B, BL18U1 and BL19U1 at the Shanghai Synchrotron Radiation Facility (SSRF) using Finback^33^, Maxcube^34^ and Bluice^35^ software. The datasets were processed using HKL2000 (HKL Research) and the XDS program^36^. The phase of the Clivia–NBSI structure was solved with the single-wavelength anomalous diffraction (SAD) method. The sites of Ir(NH_3_)_6_^3+^ were determined by SHELXC/D (see the arrow in Supplementary Fig. 4). Phasing and density modification were performed in the Crank-2 pipeline^37,38^. Iterative cycles of model building and refinement were carried out using PHENIX^39^ and Coot^40,41^. The initial model was refined with phenix.refine^39^ and adjusted manually in Coot^40,41^. Then, the structures of the Clivia–NBSI analog complexes and Clivia_III–NBSI were solved by the molecular replacement (MR) method using the Clivia–NBSI structure as the model in Phaser^42^. The ligand structures and restraints of NBSI and its analogs were generated in eLBOW from the PHENIX suite^39^. All ligands, metal ions and their coordinated waters were identified on the basis of 2*F*_o_ − *F*_c_ and *F*_o_ − *F*_c_ maps guided by their coordination geometries. The X-ray statistics of all crystals are listed in Supplementary Table 1.

### Substitutions and functional assays

The Clivia aptamer variants were transcribed using a T7 RNA polymerase transcription reaction. A phenol–chloroform extraction followed by ethanol precipitation was used to purify the transcribed aptamer. Unless otherwise indicated, all in vitro RNA characterizations were performed in 40 mM HEPES pH 7.4, 100 mM KCl and 5 mM MgCl_2_ buffer using a Synergy Neo2 microplate reader (BioTek). To test the ability of the aptamer variants to activate the fluorophore, 1 μM fluorophore was incubated with 5 μM aptamer. To calculate the dissociation constants of the fluorophore with different RNA variants, we performed a titration of 20 nM RNA aptamer with increasing concentrations of the fluorophore and then fitted the resulting data points to a curve based on the Hill equation. To test the effects of different ions on the RNA–fluorophore complex, 1 μM fluorophore was incubated with 5 μM aptamer in 40 mM HEPES (pH 7.4) and 20 mM MnCl_2_, 20 mM BaCl_2_, 20 mM CaCl_2_, 20 mM MgCl_2_ or 1 M KCl. Before the measurement, the RNAs were incubated with 100 μM EDTA and denatured at 75 °C for 10 min.

### DNA cloning, cell culture and transfection

For mammalian expression of different tandem arrays of Clivia, template DNAs encoding different copies of Clivia arrays embedded in an F30 scaffold^43^ were synthesized by Shanghai Generay Biotech. The template DNAs were amplified and inserted into the original single-guide RNA (sgRNA) expression plasmid^44^ using the pEASY-Basic Seamless Cloning and Assembly Kit (TransGen Biotech), which was linearized by PCR amplification to remove the native sgRNA scaffold. The plasmids were verified through sequencing by Shanghai Jie Li Biotech. HEK293T cells were cultured in DMEM (high glucose) supplemented with 10% FBS, incubated at 37 °C in a humidified atmosphere of 95% air and 5% CO_2_ and split every 2 days or at confluence. The cell line was authenticated and mycoplasma negative. Unless indicated, transient transfection was performed using the Hieff Trans liposomal transfection reagent (Yeasen) according to the manufacturer’s protocol. Briefly, the cells were seeded at 70–90% confluence before transfection. For a 96-well plate, 0.1 μg of DNA and 0.25 μl of Hieff Trans liposomal transfection reagent were diluted in 25 μl of Opti-MEM (Gibco). The mixture was added to the well after a 20-min incubation at room temperature.

### Fluorescence spectrometry

First, 200 µM NBSI was dissolved in 40 mM HEPES pH 7.4, 125 mM KCl, 5 mM MgCl_2_ and 24% DMSO buffer. A final concentration of 0.5–5 µM RNA was prepared in annealing buffer containing 40 mM HEPES pH 7.4, 125 mM KCl and 5 mM MgCl_2_ and annealed at 65 °C for 5 min, followed by incubation on ice for 30 min. The fluorophore ligand NBSI was added to the annealed RNAs and incubated at room temperature for another 30 min.

The 3D fluorescence spectra of individual NBSI or NBSI–RNA complexes were measured using the Cary Eclipse fluorescence spectrometer. The 3D synchronous scanning mode was selected to keep a fixed wavelength difference (*Δ*) between the excitation wavelength and the emission wavelength. The excitation wavelength range was 300–700 nm. The initial difference (*Δ*_start_) between the emission wavelength and excitation wavelength was 10 nm, the increment in wavelength difference (*Δ*_increment_) was 5 nm and the maximum wavelength difference (*Δ*_stop_) was 200 nm. The scan speed was 600 nm min^−1^ with excitation and emission slits of 5 nm.

### Imaging

For live-cell imaging of the different tandem arrays of Clivia in HEK293T cells, plasmids expressing different tandem arrays of Clivia or Broccoli were transfected into HEK293T cells. Following transfection, the cells were incubated with 1 μg ml^−1^ Hoechst 33342 and 0.2 μM NBSI, 0.2 μM NBSI571 or 10 μM DFHBI. Fluorescence imaging was performed using a Yokogawa CSU-W1 SoRa spinning disc confocal attached to an inverted Nikon (TI-E) microscope with a Nikon Perfect Focus system, a Plan Apo VC ×100/1.40 oil objective and a photometrics Prime 95B sCMOS (scientific complementary metal–oxide–semiconductor) camera, using a 405-nm laser excitation for Hoechst 33342 and a 561-nm laser excitation for Clivia.

### Reporting summary

Further information on research design is available in the Nature Portfolio Reporting Summary linked to this article.

## Online content

Any methods, additional references, Nature Portfolio reporting summaries, source data, extended data, supplementary information, acknowledgements, peer review information; details of author contributions and competing interests; and statements of data and code availability are available at 10.1038/s41589-024-01633-1.

## Supplementary information

Supplementary Information Supplementary Tables 1–3 and Figs. 1–4.

Reporting Summary

## Source data

Source Data Fig. 3 Statistical source data.

Source Data Fig. 4 Statistical source data.

Source Data Fig. 5 Statistical source data.

Source Data Fig. 6 Statistical source data.

Source Data Extended Data Fig. 3 Statistical source data.

Source Data Extended Data Fig. 4 Statistical source data.

Source Data Extended Data Fig. 5 Statistical source data.

Source Data Extended Data Fig. 7 Statistical source data.

Source Data Extended Data Fig. 9 Statistical source data.

Source Data Extended Data Fig. 10 Statistical source data.

## Data Availability

The atomic coordinates and structure factors were deposited to the PDB (www.rcsb.org) under the following accession codes: 8HZE for the Clivia–NBSI complex, 8HZJ for the Clivia–NBSI571 complex, 8HZF for the Clivia–NBSI565 complex, 8HZD for the Clivia–NBSI618 complex, 8HZK for the Clivia–NBSI complex crystals soaked with Ir(NH3)_6_^3+^, 8HZM for the Clivia–NBSI complex crystals soaked with manganese and 8HZL for the Clivia_III–NBSI complex. Source data are provided with this paper.
