# Supplementary material for: Structural basis of a small monomeric Clivia fluorogenic RNA with a large Stokes shift
Source: Nat Chem Biol. 2024 May 30;20(11):1453–60. doi: 10.1038/s41589-024-01633-1 (PMC11511665; doi:10.1038/s41589-024-01633-1)
Supplement: Supplementary file 1 — Supplementary Tables 1–3 and Figs. 1–4. [file 41589_2024_1633_MOESM1_ESM.pdf]

nature chemical biology

Supplementary information https://doi.org/10.1038/s41589-024-01633-1

Structural basis of a small monomeric Clivia fluorogenic RNA with a large Stokes shift

In the format provided by the authors and unedited

Contents

Supplementary Tables

Supplementary Table 1…………………………………………………………………….. **2**

Supplementary Table 2…………………………………………………………………….. **3**

Supplementary Table 3…………………………………………………………………….. **4**

Supplementary Figures

Supplementary Figure 1…………………………………………………………………… **5**

Supplementary Figure 2…………………………………………………………………… **6**

Supplementary Figure 3…………………………………………………………………… **7**

Supplementary Figure 4…………………………………………………………………… **8**

Supplementary Table 1 | Crystallographic statistics of Clivia aptamer related complex structure bound with NBSI

	Clivia/NBSI Native	Clivia/NBSI $[Ir(NH_3)_6]^{3+}$	Clivia/NBSI Mn^{2+}	Clivia_III/NBSI
**Data collection**				
Space group	*C*2	*C*2	*C*2	$P2_1$
Cell dimensions				
a, b, c (Å)	76.2, 45.9, 57.1	74.9, 44.0, 58.3	77.7, 46.9, 56.7	64.6, 53.9, 81.8
α, β, γ (°)	90.0, 115.1, 90.0	90.0, 112.9, 90.0	90.0, 113.7, 90.0	90.0, 105.9, 90.0
Wavelength (Å)	0.979	1.102	1.240	0.979
Resolution (Å)	50.00-1.60 (1.66-1.60)[a]	50.00-2.00 (2.07-2.00)	50.00-2.50 (2.54-2.50)	50.00-2.60 (2.69-2.60)
R_{pim}[b]	0.041 (0.237)	0.070 (0.120)	0.049 (0.329)	0.074 (0.555)
$I / \sigma I$	33.4 (3.1)	29.2 (15.9)	18.3 (1.3)	11.0 (1.5)
Completeness(%)	100 (97.1)	99.6 (99.7)	97.1 (78.1)	99.8 (100.0)
Redundancy	6.7 (6.9)	6.5 (6.6)	5.9 (3.2)	3.2 (3.3)
$CC_{1/2}$	1.00 (0.89)	0.96 (0.91)	0.98 (0.62)	0.98 (0.52)
**Refinement**				
Resolution (Å)	38.19-1.59	37.11-2.00	39.14-2.70	26.24-2.60
No. reflections	23806	11889	5218	15689
R_{work} / R_{free} (%)	18.9/21.8	20.2/24.7	18.8/24.7	18.2/24.5
No. of atoms	1792	1821	1681	3900
RNA	1572	1546	1564	3602
Ligand	54	54	54	162
Cations	14	106	16	23
Water	152	101	47	113
Others		14		
B-factors (Å^2)				
RNA	36.1	25.0	46.6	40.9
Ligand	37.6	23.1	48.2	41.5
Cations	47.2	36.6	62.9	45.7
Water	41.0	29.2	41.7	37.9
Others		25.3		
R.M.S.deviations				
Bond length (Å)	0.009	0.005	0.006	0.005
Bond angles (°)	1.15	1.08	1.16	1.08

[a]Values in parentheses are for highest-resolution shell.

[b]R_{pim}: precision-indicating merging R factor.

Supplementary Table 2 | Crystallographic statistics of Clivia aptamer structure bound with NBSI derivatives

	Clivia/NBSI571	Clivia/NBSI565	Clivia/NBSI618
**Data collection**			
Space group	*C2*	*C2*	*C2*
Cell dimensions			
a, b, c (Å)	89.3, 48.2, 58.3	84.6, 47.2, 56.2	77.5, 46.5, 56.2
α, β, γ (°)	90.0, 116.0, 90.0	90.0, 115.3, 90.0	90.0, 113.8, 90.0
Wavelength (Å)	1.102	1.102	1.102
Resolution (Å)	50.00-2.60 (2.64-2.60)[a]	50.00-2.70 (2.80-2.70)	38.86-1.87 (1.97-1.87)
R_{pim} [b]	0.033 (0.191)	0.065 (0.528)	0.026 (0.279)
$I / \sigma I$	17.1 (2.4)	10.3 (1.0)	14.4 (2.6)
Completeness(%)	98.6 (96.1)	98.9 (99.6)	96.9 (99.9)
Redundancy	6.7 (6.3)	6.7 (6.7)	6.4 (3.3)
$CC_{1/2}$	1.00 (0.93)	1.01 (0.74)	1.00 (0.89)
**Refinement**			
Resolution (Å)	29.4-2.60	40.20-2.70	28.16-1.87
No. reflections	6825	5507	14158
R_{work} / R_{free} (%)	19.2/25.8	18.2/25.4	21.1/26.0
No. of atoms	1647	1634	1697
RNA	1564	1564	1551
Ligand	50	52	54
Cations	8	6	7
Water	25	12	85
B-factors (Å^2)			
RNA	42.0	53.7	38.5
Ligand	42.7	52.2	39.1
Cations	47.4	51.8	40.6
Water	39.6	46.8	37.4
R.M.S. deviations			
Bond length (Å)	0.005	0.006	0.005
Bond angles (°)	1.08	1.36	1.04

[a]Values in parentheses are for highest-resolution shell.

[b]R_{pim}: precision-indicating merging R factor.

Supplementary Table 3 | RNA constructs used in the study

#	Name	RNA sequence, 5' to 3'
1	R8 aptamer	GGAAGAUUGUAAACAUGCCGAAAGGCAGACACUUCC
2	M1(delete U7)	GGAAGAUGUAAACAUGCCGAAAGGCAGACACUUCC
3	U7A	GGAAGAAUGUAAACAUGCCGAAAGGCAGACACUUCC
4	U7C	GGAAGACUGUAAACAUGCCGAAAGGCAGACACUUCC
5	U7G	GGAAGAGUGUAAACAUGCCGAAAGGCAGACACUUCC
6	M2 (A11G/A12G/A13G)	GGAAGAUUGUGGGCAUGCCGAAAGGCAGACACUUCC
7	M3 (A11C/A12C/A13C)	GGAAGAUUGUCCCCAUGCCGAAAGGCAGACACUUCC
8	M4 (A11U/A12U/A13U)	GGAAGAUUGUUUUCAUGCCGAAAGGCAGACACUUCC
9	M5 (U8C/A29G)	GGAAGAUCGUAAACAUGCCGAAAGGCAGGCACUUCC
10	M6 (U8A/A29U)	GGAAGAUAGUAAACAUGCCGAAAGGCAGUCACUUCC
11	M7 (A15G/G28A)	GGAAGAUUGUAAACGUGCCGAAAGGCAAACACUUCC
12	M8 (G28U)	GGAAGAUUGUAAACAUGCCGAAAGGCAUACACUUCC
13	M9 (A15C)	GGAAGAUUGUAAACCUGCCGAAAGGCAGACACUUCC
14	M10 (delete 2bp in P1)	GAGAUUGUAAACAUGCCGAAAGGCAGACACUC
15	M11 (add 2bp in P1)	GGGGAAGAUUGUAAACAUGCCGAAAGGCAGACACUUCCCC
16	M12 (delete 2bp in P2)	GGAAGAUUGUAAACACCGAAAGGGACACUUCC
17	M13 (add 2bp in P2)	GGAAGAUUGUAAACAUGCCGCGAAAGCGGCAGACACUUCC
18	II_R8	GGAAGAUUGUAAACAGCGAGAUUGUAAACAUGCCGAAAGG CAGACACUCGCGACACUUUC
19	III_R8	GGAAGAUUGUAAACAGCGAGAUUGUAAACAGCGAGAUUGU AAACAUGCCGAAAGGCAGACACUCGCGACACUCGCGACACU UUC
20	IV_R8	GGAAGAUUGUAAACAGCGAGAUUGUAAACAGCGAGAUUGU AAACAGCGAGAUUGUAAACAUGCCGAAAGGCAGACACUCGC GACACUCGCGACACUCGCGACACUUUC
21	V_R8	GGAAGAUUGUAAACAGCGAGAUUGUAAACAGCGAGAUUGU AAACAGCGAGAUUGUAAACAGCGAGAUUGUAAACAUGCCGA AAGGCAGACACUCGCGACACUCGCGACACUCGCGACACUC GCGACACUUUC
22	VI_R8	GGAAGAUUGUAAACAGCGAGAUUGUAAACAGCGAGAUUGU AAACAGCGAGAUUGUAAACAGCGAGAUUGUAAACAGCGAGA UUGUAAACAUGCCGAAAGGCAGACACUCGCGACACUCGCG ACACUCGCGACACUCGCGACACUCGCGACACUUUC

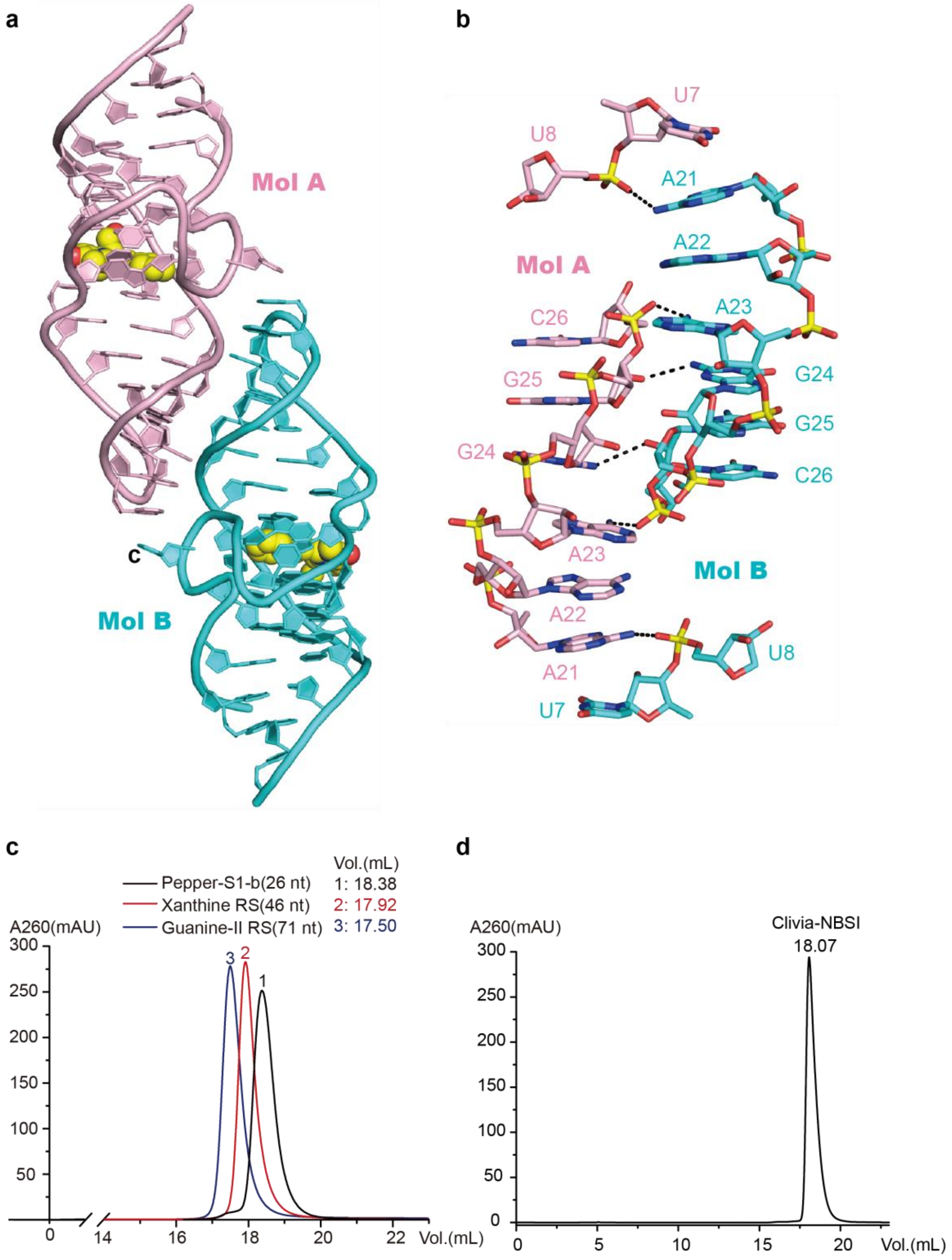

Supplementary Figure 1 | Packing alignment and the intermolecular interactions in the crystal lattice.

(**a**) Each asymmetric unit of the Clivia-NBSI crystal structure contains two molecules, Mol A (in pink) and Mol B (in cyan). Both of them are shown in cartoon.

(**b**) U7 protrudes from the helical structure of one molecule and is partially stacked with the variable loop of stem P2 of the other molecule in the asymmetric unit. Hydrogen bonding interaction are also formed between the two molecules (Mol A and Mol B).

(**c, d**) Size-exclusion experiments were conducted using a Superose™ Increase 10/300 GL column to determine the solution state of Clivia. Three monomeric RNA molecules of varying lengths—26 nt, 46 nt, and 71 nt—were analyzed to serve as references for this purpose (**c**). The running buffer contains 50 mM HEPES, pH 6.8, 50 mM NaCl and 5 mM $MgCl_2$. Clivia exists homogenously as monomer in solution (**d**).

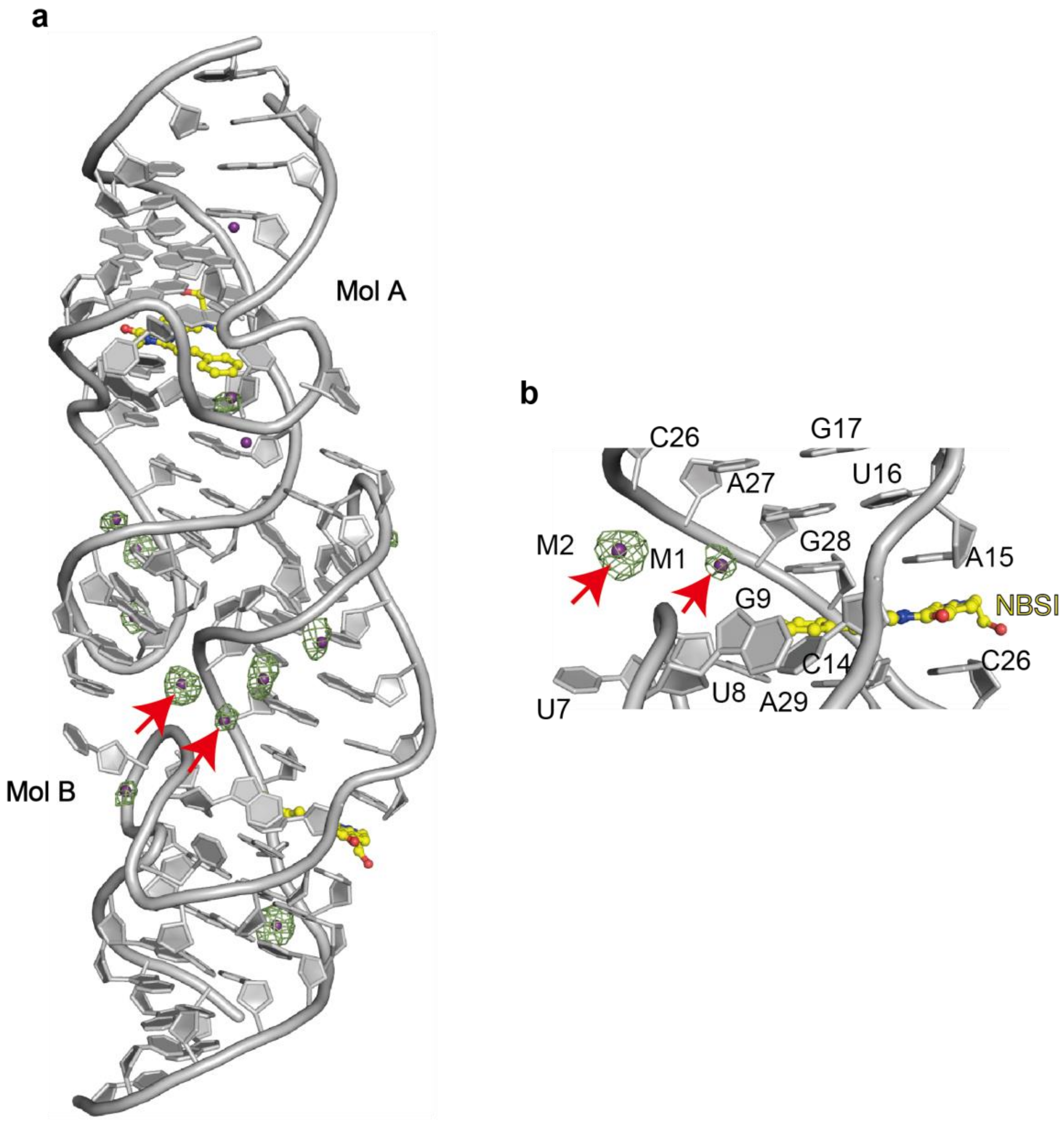

Supplementary Figure 2 | Packing alignment in the crystal lattice and the bound Mn^{2+} sites in Clivia-NBSI structure.

(**a, b**) Anomalous map of the Mn^{2+}-soaked Clivia-NBSI crystal was calculated and contoured at level 3.0 σ to show the signal of the substituted Mn^{2+} ions. The sites M1 and M2 are indicated with red arrows.

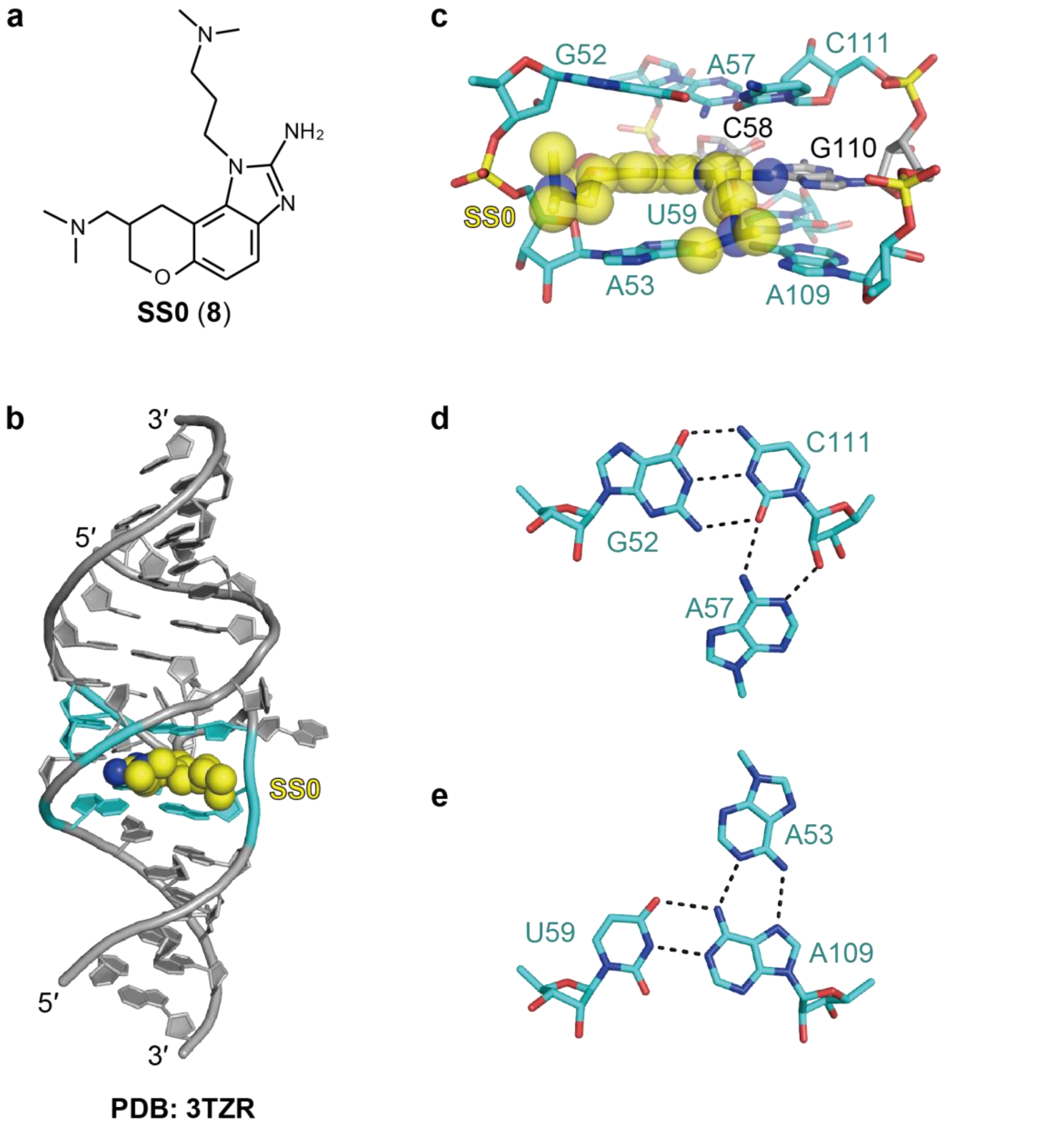

Supplementary Figure 3 | Structure of RNA structure from the Internal Ribosome Entry Site of Hepatitis C Virus.

(**a**) Chemical structure of the bound ligand SS0 (**8**).

(**b**) Tertiary structure of riboswitch-like RNA structure from the Internal Ribosome Entry Site of Hepatitis C Virus (PDB: 3TZR).

(**c-e**) The composition of the binding pocket of ligand SS0 (**8**). ligand SS0 (**8**) was recognized by G52-C111-A57 (**d**) and U59-A109-A53 base triples (**e**).

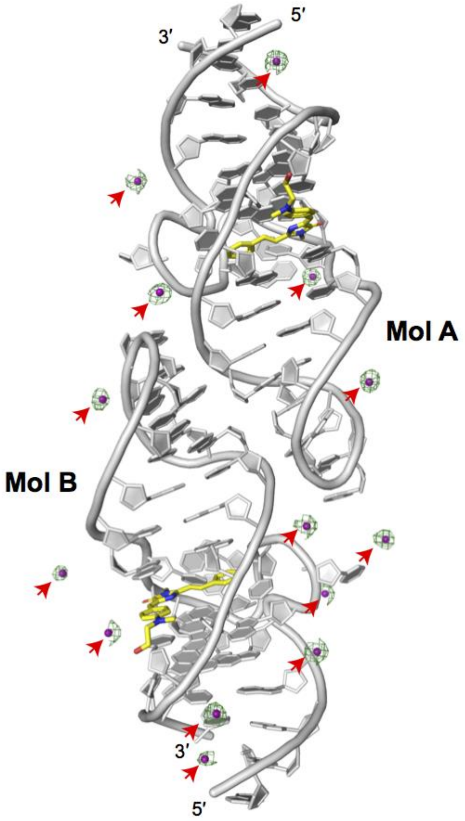

Supplementary Figure 4 | Bound $Ir(NH_3)_6^{3+}$ sites in structure of Clivia aptamer. Bound $Ir(NH_3)_6^{3+}$ sites (shown by red arrows) involved in the same crystal lattice with anomalous map contoured at 3.0 σ level.
