# Supplementary material for: Structural basis of a small monomeric Clivia fluorogenic RNA with a large Stokes shift
Source: Nat Chem Biol. 2024 May 30;20(11):1453–60. doi: 10.1038/s41589-024-01633-1 (PMC11511665; doi:10.1038/s41589-024-01633-1)
Supplement: Supplementary file 2 — Reporting Summary [file 41589_2024_1633_MOESM2_ESM.pdf]

nature research

Corresponding author(s): Aiming Ren

Last updated by author(s): Apr 5, 2024

Reporting Summary

Nature Research wishes to improve the reproducibility of the work that we publish. This form provides structure for consistency and transparency in reporting. For further information on Nature Research policies, see our Editorial Policies and the Editorial Policy Checklist.

Statistics

For all statistical analyses, confirm that the following items are present in the figure legend, table legend, main text, or Methods section.

n/a	Confirmed	
☐	☒	The exact sample size (*n*) for each experimental group/condition, given as a discrete number and unit of measurement
☐	☒	A statement on whether measurements were taken from distinct samples or whether the same sample was measured repeatedly
☒	☐	The statistical test(s) used AND whether they are one- or two-sided<br>*Only common tests should be described solely by name; describe more complex techniques in the Methods section.*
☒	☐	A description of all covariates tested
☐	☒	A description of any assumptions or corrections, such as tests of normality and adjustment for multiple comparisons
☐	☒	A full description of the statistical parameters including central tendency (e.g. means) or other basic estimates (e.g. regression coefficient) AND variation (e.g. standard deviation) or associated estimates of uncertainty (e.g. confidence intervals)
☒	☐	For null hypothesis testing, the test statistic (e.g. *F*, *t*, *r*) with confidence intervals, effect sizes, degrees of freedom and *P* value noted<br>*Give P values as exact values whenever suitable.*
☒	☐	For Bayesian analysis, information on the choice of priors and Markov chain Monte Carlo settings
☒	☐	For hierarchical and complex designs, identification of the appropriate level for tests and full reporting of outcomes
☒	☐	Estimates of effect sizes (e.g. Cohen's *d*, Pearson's *r*), indicating how they were calculated

Our web collection on statistics for biologists contains articles on many of the points above.

Software and code

Policy information about availability of computer code

Data collection	Descriptions of all the data collection are provided in the method section. The X-ray diffraction data were collected at the National Facility for Protein Science in Shanghai (NFPS), Zhangjiang Lab, China, using Finback, Maxcube and Bluice softwares. Fluorescence imaging was performed using a Yokogawa CSU-W1 SoRa spinning disk confocal attached to an inverted Nikon (TI-E) microscope with Nikon Perfect Focus system, a Plan Apo VC 100×/1.40 oil objective, a photometrics Prime 95B sCMOS camera.
Data analysis	Descriptions of all the data analysis and the used software are provided in the method section and the supplementary materials. The datasets were processed by HKL2000 (HKL Research) and XDS program. The structures were solved and refined with SHELXC/D, Crank-2 pipeline, PHENIX, Phaser and coot programs. Imagel 1.49k was used to process the imaging data.

For manuscripts utilizing custom algorithms or software that are central to the research but not yet described in published literature, software must be made available to editors and reviewers. We strongly encourage code deposition in a community repository (e.g. GitHub). See the Nature Research guidelines for submitting code & software for further information.

Data

Policy information about availability of data

All manuscripts must include a data availability statement. This statement should provide the following information, where applicable:

- Accession codes, unique identifiers, or web links for publicly available datasets
- A list of figures that have associated raw data
- A description of any restrictions on data availability

The atomic coordinates and structure factors have been deposited with the Protein Data bank (www.rcsb.org) under the following accession codes: 8HZE for Clivia-NBSI complex, 8HZJ for Clivia-NBSI571 complex, 8HZF for Clivia-NBSI565 complex, 8HZD for Clivia-NBSI618 complex, and 8HZK for Clivia-NBSI complex crystals soaked with Ir(NH3)63+, 8HZM for Clivia-NBSI complex crystals soaked with manganese and 8HZL for Clivia_III-NBSI complex.

Field-specific reporting

Please select the one below that is the best fit for your research. If you are not sure, read the appropriate sections before making your selection.

☒ Life sciences ☐ Behavioural & social sciences ☐ Ecological, evolutionary & environmental sciences

For a reference copy of the document with all sections, see nature.com/documents/nr-reporting-summary-flat.pdf

Life sciences study design

All studies must disclose on these points even when the disclosure is negative.

Sample size	The sample sizes chosen for each experiment were indicated in the method section or figure legends.
Data exclusions	Some diffraction outliers of the crystals were automatically excluded by the established program according to the criteria.
Replication	All the attempts to replicate the experiments were successful.
Randomization	Samples were randomly distributed into groups.
Blinding	The authors were not aware of the results before performing the experiments.

Reporting for specific materials, systems and methods

We require information from authors about some types of materials, experimental systems and methods used in many studies. Here, indicate whether each material, system or method listed is relevant to your study. If you are not sure if a list item applies to your research, read the appropriate section before selecting a response.

Materials & experimental systems

n/a	Involved in the study	
☒	☐	Antibodies
☐	☒	Eukaryotic cell lines
☒	☐	Palaeontology and archaeology
☒	☐	Animals and other organisms
☒	☐	Human research participants
☒	☐	Clinical data
☒	☐	Dual use research of concern

Methods

n/a	Involved in the study	
☒	☐	ChIP-seq
☒	☐	Flow cytometry
☒	☐	MRI-based neuroimaging

Eukaryotic cell lines

Policy information about cell lines

Cell line source(s)	HEK293T (GNHu44) was purchased from the Cell Bank of Chinese Academy.
Authentication	No cell lines were authenticated.
Mycoplasma contamination	The cell line has been tested negative for mycoplasma containimation by PCR methods.
Commonly misidentified lines (See ICLAC register)	No commonly misidentified cell lines were used.
